# Comparative Profiling of miRNAs and Target Gene Identification in Distant-Grafting between Tomato and *Lycium* (Goji Berry)

**DOI:** 10.3389/fpls.2016.01475

**Published:** 2016-10-18

**Authors:** A. B. M. Khaldun, Wenjun Huang, Haiyan Lv, Sihong Liao, Shaohua Zeng, Ying Wang

**Affiliations:** ^1^Key Laboratory of Plant Germplasm Enhancement and Specialty Agriculture, Wuhan Botanical Garden (CAS)Wuhan, China; ^2^University of the Chinese Academy of SciencesBeijing, China; ^3^Oilseed Research Center, Bangladesh Agricultural Research Institute (BARI)Joydebpur, Gazipur, Bangladesh; ^4^Key Laboratory of South China Agricultural Plant Molecular Analysis and Genetic Improvement, Provincial Key Laboratory of Applied Botany, South China Botanical Garden (CAS)Guangzhou, China; ^5^Northwest Center for Agrobiotechnology (Ningxia), CASBeijing, China

**Keywords:** *Lycium*, tomato, distant-grafting, miRNA, small RNA

## Abstract

Local translocation of small RNAs between cells is proved. Long distance translocation between rootstock and scion is also well documented in the homo-grafting system, but the process in distant-grafting is widely unexplored where rootstock and scion belonging to different genera. Micro RNAs are a class of small, endogenous, noncoding, gene silencing RNAs that regulate target genes of a wide range of important biological pathways in plants. In this study, tomato was grafted onto goji (*Lycium chinense* Mill.) to reveal the insight of miRNAs regulation and expression patterns within a distant-grafting system. Goji is an important traditional Chinese medicinal plant with enriched phytochemicals. Illumina sequencing technology has identified 68 evolutionary known miRNAs of 37 miRNA families. Moreover, 168 putative novel miRNAs were also identified. Compared with control tomato, 43 (11 known and 32 novels) and 163 (33 known and 130 novels) miRNAs were expressed significantly different in shoot and fruit of grafted tomato, respectively. The fruiting stage was identified as the most responsive in the distant-grafting approach and 123 miRNAs were found as up-regulating in the grafted fruit which is remarkably higher compare to the grafted shoot tip (28). Potential targets of differentially expressed miRNAs were found to be involved in diverse metabolic and regulatory pathways. ADP binding activities, molybdopterin synthase complex and RNA helicase activity were found as enriched terms in GO (Gene Ontology) analysis. Additionally, “metabolic pathways” was revealed as the most significant pathway in KEGG (Kyoto Encyclopedia of Genes and Genomes) analysis. The information of the small RNA transcriptomes that are obtained from this study might be the first miRNAs elucidation for a distant-grafting system, particularly between goji and tomato. The results from this study will provide the insights into the molecular aspects of miRNA-mediated regulation in the medicinal plant goji, and in grafted tomato. Noteworthy, it would provide a basis how miRNA signals could exchange between rootstock and scion, and the relevance to diverse biological processes.

## Introduction

Grafting is considered as one of the most successful techniques for plant and crop production, and protection. Literally, grafting is the union of a bud or stem or even a branch onto the root or stem or branch of another plant, while it is termed as a “distant-grafting” when it is carried out among different families, genus or species.

In recent years, distant-graft mutagenesis technology developed instantaneously and proved its ability to bypass the natural obstacles of incompatibility between distantly-related species. Notably, to integrate the rootstock of different sources and scion organically thus allow the scion to grow, develop and fruit normally with desired traits (Pan et al., 2012). Though it was thought initially that the genetic materials of rootstock integrate into the scion's genome creates the genetic variation induced by grafting (Taller et al., 1998), however, recent molecular biology research has identified that the stress-related retro-transposons have undergone transposition due to distant-grafting mechanism (Wei-Min, 2005), which is recognized as one of the effective mechanisms of genome rearrangement and gene mutation. Similarly, some other studies confirmed that the nucleic acid materials of rootstock can transmit through the graft-junction to the scion, where small RNA might silence, some special genes function or regulate expression of genes in scion (Stegemann and Bock, 2009). Undoubtedly, grafting confers a new physiological event to the involved plants though the molecular processes are relatively unknown. The gene expression changes occurred at transcriptional, post-transcriptional, and post-translational levels in the grafted plants. However, the mechanism of gene or loci expression regulation and the affiliation of small RNAs are largely unknown.

MicroRNAs (miRNAs) is a recently discovered small RNA class, which is short (20–24 nt), endogenous and non-coding, that regulates gene expression at post transcriptional level by cleaving or inhibiting translation of target gene transcripts (Llave et al., 2002; Joshi et al., 2010). Recent evidence indicates that they play a critical and pivotal role in the most significant biological processes and molecular pathways such as plant growth and development, including leaf morphology and polarity, lateral root formation, hormone signaling, transition between vegetative stage to flowering stage, regulation at flowering time, floral organ identity, and reproduction (Mallory and Vaucheret, 2006; Sunkar et al., 2007). Additionally, they have regulatory effects on nutrition homeostasis, abiotic stresses, and pathogen responses (Carrington and Ambros, 2003; Song et al., 2010). In plant, miRNAs are transcribed by RNA polymerase II to produce pri-miRNA (primary miRNA) transcripts and been cleaved to pre-miRNAs (miRNA precursors) followed by the catalytic activity of DCL1 (Dicer-like enzyme) (Subramanian et al., 2008; Naqvi et al., 2012). The pre-miRNA is additionally cleaved to a miRNA duplex (miRNA:miRNA^*^) and then one of these strand is incorporated into RISC (RNA induced silencing complex) (Szittya et al., 2008). Usually miRNA^*^ is degraded, but also can be accumulated at a lower level in some exceptions (Wang et al., 2011). Then the remaining mature miRNA sequence guides AGO (Argonaute protein) toward the complementary target mRNA sequence resulting the silencing of the corresponding genes (Bartel, 2004; Zhu, 2008).

Recent studies of long-distance signal movement and functional analysis have proven that the macromolecules like proteins, mRNAs and miRNAs can translocate through the phloem. Moreover, these entities act as signals for the stress-related pathways, and for plant development (Kehr and Buhtz, 2008; Atkins et al., 2011). FLOWERING TIME protein is a well-studied molecule that moves from leaves to shoot apex in *Arabidopsis* (Corbesier et al., 2007). In few cases, movements of siRNAs (small interfering RNAs) were reported where induction of posttranscriptional gene silencing acted against viruses (Waterhouse et al., 2001). Previous report of biochemical analysis in pumpkin (*Cucurbita maxima*), has characterized a unique component of protein machinery CmPSRP1 (Phloem SMALL RNA BINDING PROTEIN1), which selectively binds to the small single-stranded RNA (ssRNA) species and mediates cell-to-cell trafficking, but not with dsRNA molecules (Hamilton et al., 2002). Further molecular study revealed that the endogenous CmPP16 moved from CC (companion cell) into SE (sieve element) through the pore-plasmodesmata when grafting was performed between pumpkin and cucumber (Tiedemann and Carstensbehrens, 1994).

Many studies reported on grafting, but very limited information is available on plant miRNAs mobility. Some other reviews have summarized presence of miRNAs in phloem exudates of different species, but negligible information reported on their mobility (Chuck and O'Connor, 2010; Kehr and Buhtz, 2013). However, RNA silencing signal movement was first convincingly reported in tobacco experiments (Palauqui et al., 1997). In a report of root patterning, miRNA165/166 appeared to move to adjacent cell layers from its biogenesis site. This is an example of a short-distance movement of miRNAs (Carlsbecker et al., 2010; Miyashima et al., 2011). However, recent evidence has indicated that only four miRNAs (miR399, miR395, miR172, and miR156) have been demonstrated as long-distance mobile signals in plants (Bhogale et al., 2014). Pant et al. (2008) reported that miR399 acted as a movable signal in *Arabidopsis* which regulated the phosphate homeostasis (Pant et al., 2008), whereas miR395 was reported to move from wild-type scions to rootstocks miRNA processing mutant *hen1-1* under sulfate stress (Buhtz et al., 2010). In another study, miR172 was proposed as a long-distance mobile signal responsible for tuberization in potato (*Solanum tuberosum*) (Martin et al., 2009). A recent report by Bhogale et al. confirmed miR156 as a graft-transmissible miRNA which modulated potato plant architecture and tuberization in a potato micro-grafting method (Bhogale et al., 2014).

Our study was on grafting between tomato and goji. Both tomato (*Solanum lycopersicum* L.) and goji (*Lycium chinense* Mill.), both belonging in the night shade family (Solanaceae) of different genera. Goji, commercially called wolfberry/goji berry, is the common name to the fruit of two very closely related species: *Lycium barbarum* L. (Ningxia gouqi) and *Lycium chinense* Mill. (gouqi) (Flint, 1997). Tomato and goji are well recognized and high valued species for their unique and diversified uses and qualities. Goji fruit has a significant position at the glorious Chinese traditional medicine history.

According to the Chinese traditional medicine study, goji fruits are effective in nourishing liver and kidney, enhancing eyesight, enriching blood, invigorating sex, reducing rheumatism and so on. Some other important functions have been confirmed in modern clinical researches like immunity improvement, anti-oxidation, anti-aging, growth stimulation, hemopoiesis enhancing, incretion regulating, blood sugar reducing and bearing improvement. Goji is also widely used in brewing, beverage and many other food products (Dong et al., 2008; Zhao et al., 2013).

On the other hand, tomato is the world's top canned and the largest vegetable crop after potato and sweet potato (Fawusi, 1978). It is a very cheap source of vitamins and important condiment in the most diets. Along with tremendous invaluable nutritional functions of tomato, recent years, “lycopene” (a phytochemical), has drawn centric attention for its disease-fighting abilities, which is abundantly found in tomato. Studies in human have resulted that lycopene is effective at a wide range of cancers, including prostate of course, but also colorectal, breast, lung, endometrial, pancreatic, bladder, cervical, and skin cancers. Lycopene furthermore helpful to prevent heart disease and may slow the development of cataracts and muscular degeneration, an age-related vision problem that can lead to blindness (Agarwal and Rao, 2000).

By far the most conserved plant miRNAs have been identified through traditional Sanger sequencing method, but high-throughput sequencing technology is replacing Sanger and computational prediction methods vigorously. In computation-based approach, miRNAs can be identified with high conservation, but it cannot be characterized and likely have a probability to produce false-positive results (Xu et al., 2010). Recent studies reported that species-specific miRNAs of non-model plants are also very important. Moreover, non-conserved miRNAs are found at a low level of expression (Moxon et al., 2008). The deep sequencing technology can detect miRNAs with a high precision even less abundantly expressed miRNAs with a lower cost. Thus, miRNAs detection through small RNA library construction of high-throughput sequencing is considered as a feasible way in small RNA research (Song et al., 2010).

MiRNAs have been studying extensively, but this study might be the first miRNA elucidation of distant-grafting, particularly between medicinally important goji berry and tomato. However, miRNAs profiling from grafted tomato never been reported. Our present study focused on the miRNA-regulated gene expressions based on high-throughput small RNA libraries of grafted and control tomato samples. Obtained results would provide a unique basis for further unraveling the mechanism of miRNA transshipment between rootstock and scion and to build up the relevance to diverse biological processes between these plants.

## Materials and methods

### Plant material

*Lycium chinense* Mill. (cv. Large leave goji), was selected as a rootstock for this study and a commercial tomato cultivar, TA209 was selected as the scion. Healthy, fresh, and strong stem of goji was collected and grown in soil under control condition to develop mother stock plants. After several months, the stock plants sprouted a good number of branches and healthy leaves. Approximately, 2 months old, non-lignified and soft woody branch was chosen as rootstock. On the other hand, tomato seeds were germinated and grown under control conditions and the healthy shoot of approximately 6–7 weeks old was selected as scion.

### Grafting procedure

The stem of goji was cut about 15–20 cm above the soil level and made a vertical cut of 1–2 cm at the center. 3–5 cm stature scions were prepared by cleaning leaves and trimmed the base as a wedge followed by cleft grafting method (Sonoda and Nishiguchi, 2000). Graft junction was wrapped and covered immediate after grafting. The junction was wrapped by a polythene stripe and water was spraying three times in a day to avoid the desiccation of tender tomato shoot. Three weeks later, the bag has been removed permanently. Since the expression level of plant miRNAs changes on the spatio-temporal basis (Wang et al., 2012), we, followed the homo-grafting method in tomato to avoid the cutting/mechanical effect upon the miRNAs expression pattern. So, finally, we have produced two types of tomato plants for our small RNA study; (a) grafted tomato plant (where rootstock is goji) and (b) control tomato plant (where rootstock is a tomato itself).

### Sample tissue selection, total RNA extraction, high-throughput sequencing and small RNA library construction

For the high-throughput RNA sequencing and the small RNA library construction; we have studied four samples. Two types of samples (shoot and fruit) were studied from grafted and control tomato plants, thus four libraries were constructed such as: SFC (solanaceae fruit control); SFG (solanaceae fruit graft); SSC (solanaceae shoot control) and SSG (solanaceae shoot graft). For the shoot tissues, we have considered a shoot tip of 2 months old with the adjacent tender leaves and for fruit tissues, three fruits developmental stages (mature green, color breaking and ripening red) were considered. Target tissues were collected into sealer bags followed by snap freezing immediately in liquid N_2_ and stored into liquid N_2_ until crashing. The total RNAs of shoot were isolated from the shoot tip along with the adjacent leaves and for fruit, the total RNAs were isolated from the three fruit developing stages separately followed by the equal bulk mixing for the RNA sequencing and subsequent validation experiments. Target tissues were stored at −80°C ultra-refrigerator for later or further uses.

The total RNAs were isolated from target tissues by the TRIzol method, with a slight modification using RNAiso Plus (TAKARA BIO INC.) extraction kit, and the methods that were described previously (Hafner et al., 2008). RNA quantification and qualification were ensured before constructing small RNA libraries. One percent agarose gel electrophoresis was performed to monitor RNA degradation and contamination. Nano photometer (Implen, CA, USA) was used to check RNA purity. RNA concentration was measured using Qubit 2.0 flurometer (Life Technologies, CA, USA) and the integrity was assessed by Agilent bioanalyzer 2100 (Agilent Technologies, CA, USA). The Illumina sequencing was used at Novogene Company, Beijing, China. In short, the total RNAs were separated on 8% PAGE for small RNAs selection followed by ligation with Solexa 5′ and 3′ adapters sequentially. Adapter-ligated small RNAs were reverse transcribed after ligation and purification, finally PCR products were sequenced on an Illumina Hiseq 2500/2000 platform. The 50 bp single-end reads were generated.

### Bioinformatics analyses of sequencing data

From the sequenced, raw reads (raw data); only clean reads were used for the downstream analyses (a flowchart of the bioinformatics is included in the Figure S1). Raw reads of the fastq format were processed through Perl and python scripts. Then the reads were screened out to separate clean reads by removing contaminating reads, adapters containing sequences, insert tags, reads with poly-A tails and the sequences beyond the 18–40 nt length. Clean reads of these sRNA tags were mapped to the tomato genome (http://solgenomics.net/organism/Solanum_lycopersicum/genome) using Bowtie (Langmead et al., 2009) with no mismatches. Non-coding RNAs (rRNAs, tRNAs, snRNAs, snoRNAs) matching sequences was BLASTN searched with the sequences of Rfam (http://www.sanger.ac.uk/software/Rfam) and NCBI GenBank databases (http://www.ncbi.nlm.nih.gov/blast/Blast.cgi) and were excluded to perform the downstream analyses (Benson et al., 2011).

The remaining sRNA tags were used to search the evolutionary conserved or known miRNAs. Reads were searched against miRBase 20.0 considering maximum two mismatch (Griffiths-Jones et al., 2008). To obtain the potential miRNAs and drawing the secondary structures, a modified software, miRDeep2 (Friedlander et al., 2012) and srna-tools-cli were used. The characteristic of the hairpin structure of miRNA precursors was used to predict the potential novel miRNAs. By the integrating operation of miREvo (Wen et al., 2012) and miRDeep2 software, novel miRNAs were predicted through exploring the secondary structures, DCL1 cleavage sites and the minimum free energy (MFE) of the sRNA tags.

To predict miRNA families, we have used known miRNAs in miFam.dat (http://www.mirbase.org/ftp.shtml) and the novel miRNA precursor were aligned to Rfam (http://rfam.xfam.org/). For the prediction of potential target genes of miRNAs, psRobot software was used (Wu et al., 2012).

Quantification is very important to estimate the expression of miRNAs, so after prediction of the reads corresponding to the target genes was normalized by TPM (transcripts per million) according to Zhou et al. (2010). The normalization formula is: normalized expression = mapped read count/total reads^*^1,000,000.

For the detection of differential expression of the miRNAs between two samples, we have used DEGseq (2010) R package (to minimize the positive false rate for the sequencing data for without biological replicates). The corresponding *P*-values were adjusted using *q*-values (Storey, 2003). The threshold level of *q*-value was set as *q* < 0.01 and |log_2_(foldchange)|>1 to estimate the significantly differentially expressed miRNAs.

The dataset studied in this article is available in the NCBI (SRA) public repository under the accession number SRP051797.

### Validation of miRNA expression using qRT-PCR

Poly (A)-tailed RT-qPCR was used to measure the gene expression variation and validate the deep sequencing results of miRNAs. Reverse-transcription reaction and real-time PCR primer design were conducted according to previous report (Shi and Chiang, 2005) and the manufacturer's instructions (Takara-Primescript miRNA qPCR starter kit, 2.0), where each PCR reaction was performed in a volume of 25 μl containing 12.5 μl of SYBR Premix Ex Taq II (2×), 1 μl of each forward primer, 1 of μl universal reverse primer and reverse-transcribed cDNA from ~100 pg of total RNA. The PCR protocol was 5 s at 95°C, 40 cycles of 95°C for 5 s, 60°C for 20 s and 72°C for 1 min. Real- time PCR was performed on the Applied Biosystem 7500 Fast detection system using the SYBR Green I method, and all the reactions were run in biological triplicates. The melting curve was used to determine the specificity of PCR products (primer amplicons).

The delta-C_T_ (corresponding cycle threshold) method was used to calculate the relative expressional levels of miRNAs (Livak and Schmittgen, 2001). In this method, the C_T_ values of the wanted miRNAs and reference genes were first transformed for measurement using delta-C_T_ followed by dividing the quantities of wanted miRNAs by the geometric mean of the reference genes. The standard deviation and mean values were calculated using to triplicates RT-qPCR assays. The C_T_ was calculated using the machine accessory software and converted into relative copy numbers using a standard curve as previously described reports (Jones-Rhoades and Bartel, 2004; Shi and Chiang, 2005). The gene 5.8 S rRNA was used as reference gene in the qPCR detection of miRNAs. Student's *t*-test was used for the statistical analysis of the RT-qPCR.

### Functional assignments of potential target genes

GO enrichment analysis was used for the target gene candidates of differentially expressed miRNAs. GOseq based Wallenius non-central hyper-geometric distribution (Young et al., 2010) was used in the GO enrichment analysis. KEGG database was used for further understanding of the functional diversity of “target gene candidates” (Kanehisa et al., 2008). Finally, we have used KOBAS (Mao et al., 2005) software to test the statistical enrichment of the target gene candidates in KEGG pathways.

### Validation of predicted targets using RLM 5′-race

For the validation of candidate targets, modified RNA Ligase-Mediated 5′-RACE (RLM 5′-RACE) was performed. 5′-Full RACE Kit (Takara) was used according to the manufacturer's instructions, with slight modifications. Briefly, total RNA was directly ligated to the 5′ adaptor followed by reverse transcription with the oligo (dT) primers. PCR was performed with 5′ primers and 3′ gene-specific primers using the cDNA as the template (Table S1). The 5′-RACE PCR products were purified using Takara PCR product recovery kit, cloned, and sequenced.

## Results

### Summary of small RNAs generated through deep sequencing in grafted and control tomato plants

For the investigation of small RNAs, especially miRNAs' regulatory network in grafted tomato plant, four small RNA libraries were constructed from the shoot and fruit tissues of the control and grafted tomato plant followed by prediction of involved targets and important regulatory biological pathways. More than 35.22 million raw reads were obtained from four libraries and approximately 34.38 million (97.63%) found with high quality (Table 1).

**Table 1:** **Statistical summary of data generated by high-throughput small RNA sequencing in tomato**.

**Reads type**	**sRNA libraries**				**Total**
	**SFC**	**SFG**	**SSC**	**SSG**	
Total reads	6,825,823	10,175,215	10,309,183	7,914,429	35,224,650
Clean reads	6,653,849	9,927,920	10,077,439	7,729,107	34,388,315
Total sRNA reads	5,951,833 (100%)	9,140,022 (100%)	9,448,716 (100%)	6,494,077 (100%)	31,034,648 (100%)
Unique sRNA reads	1,049,627	3,594,206	4,536,766	3,437,092	14,617,691
Mapped sRNAs	3,677,321 (61.78%)	3,669,417 (40.15%)	6,574,077 (69.58%)	4,577,167 (70.48%)	18,497,982 (100%)

*Total reads, total number of raw reads; Clean reads, the reads remaining after low quality reads, N%> 10%, 5′ adapter contaminants, 3′ adapter null and Poly (A/T/G/C); Total sRNA reads, number of total clean sRNA reads; Unique sRNA reads, number of different categories of clean sRNA reads; Mapped sRNAs, number of different sRNA reads that are mapped to the reference genome and their ratios of total sRNA reads. SFC, Solanaceae fruit control; SFG, Solanaceae fruit graft; SSC, Solanaceae shoot control; SSG, Solanaceae shoot graft*.

**Figure 1:**
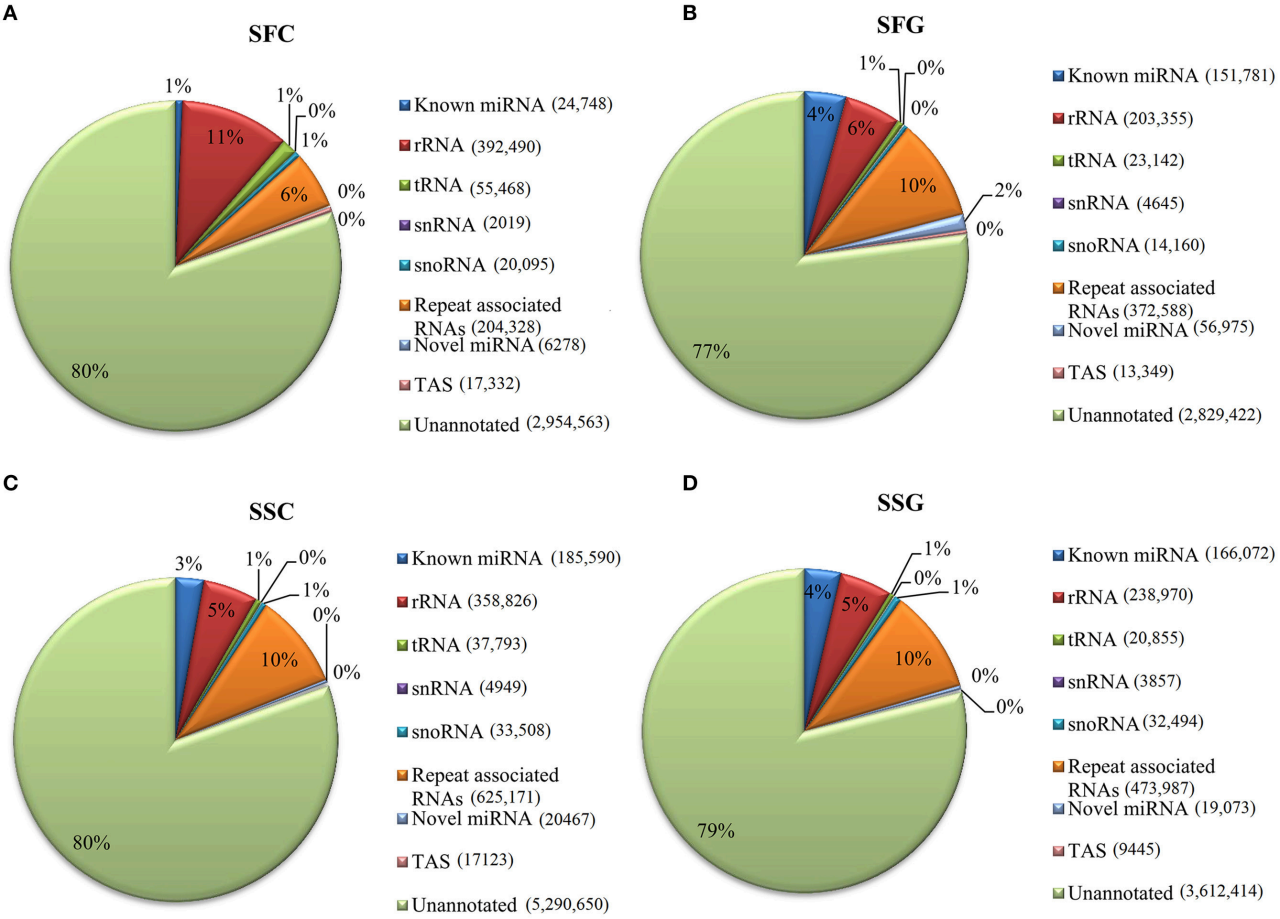
**Small RNA classification. (A)** Small RNAs in SFC library (Solanaceae fruit control); **(B)** Small RNAs in SFG (Solanaceae fruit graft); **(C)** Small RNAs in SSC (Solanaceae shoot control); **(D)** Small RNAs in SSG (Solanaceae shoot graft). Known miRNA, perfect matching mature miRNA sequences in miRBase miRNAs repository; Novel miRNA, new mature miRNA sequences detected by characteristic stem-loop hairpins and minimum free energy (MFE); rRNA, tRNA, snRNA, snoRNA are non-coding sRNAs category; TAS, transacting small interfering RNAs; Unannotated, reads that had no known information.

**Figure 2:**
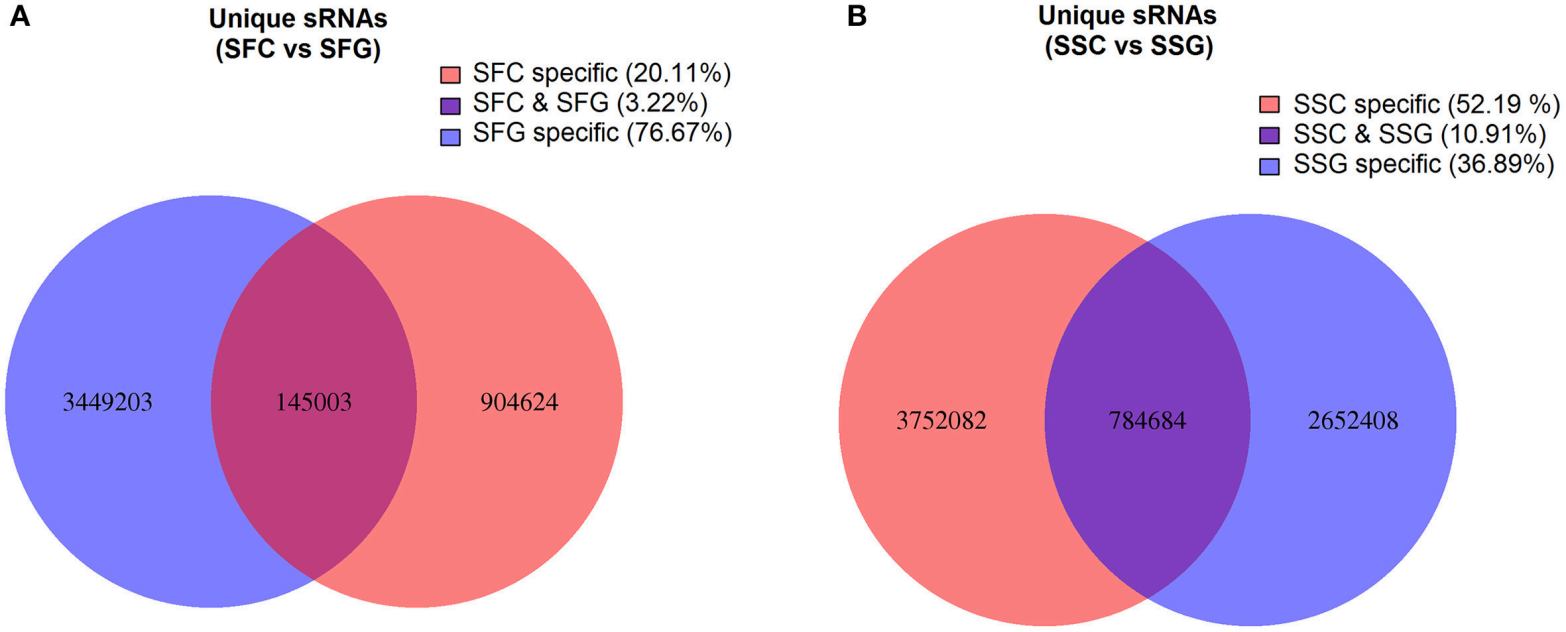
**Common and specific unique sRNA sequence in SFC vs. SFG (A) and SSC vs. SSG (B)**. SFC, Solanaceae fruit control; SFG, Solanaceae fruit graft; SSC, Solanaceae shoot control; SSG, Solanaceae shoot graft.

After removing different adapters, poly-(A/T/G/C) sequences and reads beyond the 18–40 nt range, 31,034,648 (88.10% of total raw reads) total small RNA reads with 18,497,982 mapped sRNA sequences were obtained from the four libraries (Table 1). These sequences were comprised of known miRNAs, putative novel miRNAs, rRNAs, tRNAs, snRNAs, snoRNAs, repeat associated RNAs, TAS (Transacting small interfering RNAs or TASi) and unannotated fragments (Figure 1). These total clean sRNA tags were then mapped to the tomato genome without any mismatches by Bowtie software to analyze their expression and distribution on the reference (Langmead et al., 2009). The highest proportion of reads mapped to the tomato genome found from SSG (Solanaceae shoot graft) library 4,577,167 (70.48%), and the lowest proportion yielded from SFG library 3,669,417 (40.15%). Important observation was found from the unique sRNA reads in the grafted samples. Grafted tomato fruit has shown notably higher accumulation of unique sRNA reads over control (39.32 and 17.63% of clean sRNA reads, respectively). Similarly, grafted shoot also has demonstrated exceptionally higher frequency than control (83.72 and 48.01% of clean sRNA reads, respectively; Table 1). This phenomenon strongly supports that a tremendous changes have occurred in transcriptomic level of tomato tissues from the rootstock of goji. In this study, 76.67% unique reads were found common between SFC and SFG libraries where 20.11% were SFC specific and 3.22% were SFG specific. In SSC and SSG libraries only 10.91% were common and 52.19 and 36.89% were found to be SSC and SSG specific, respectively (Figure 2).

**Figure 3:**
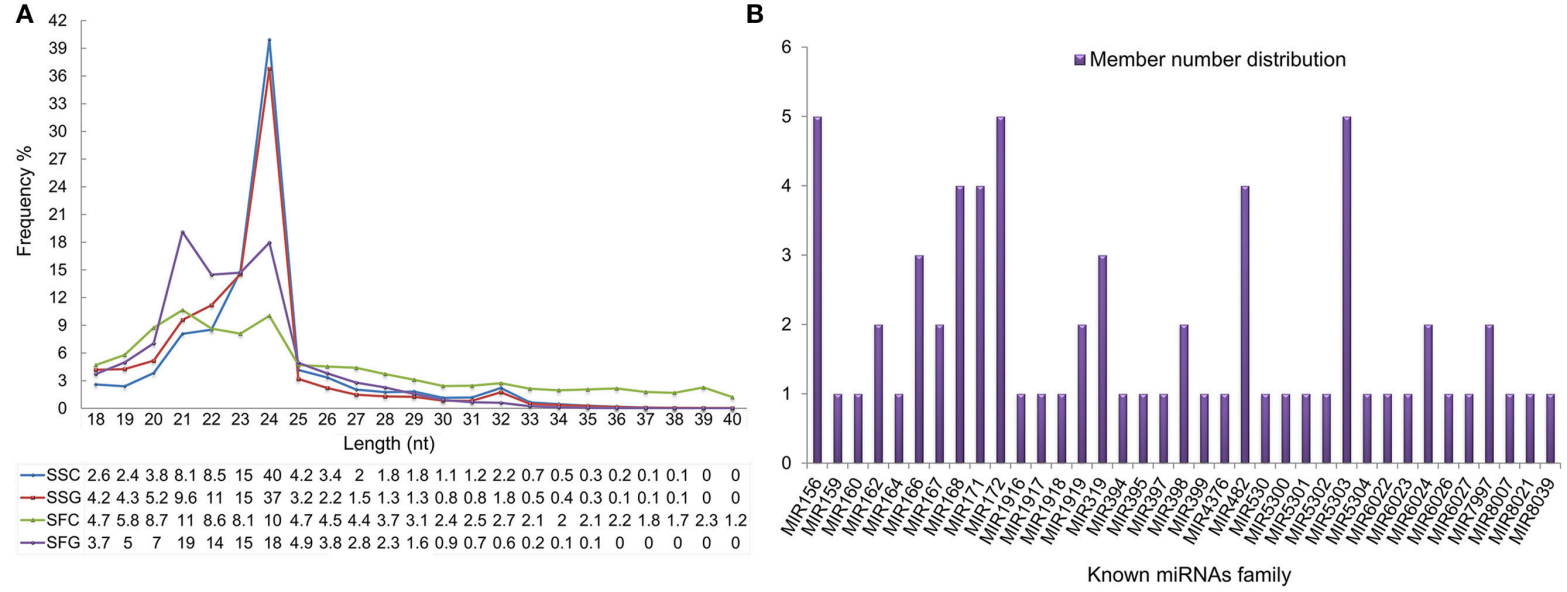
**Length distribution of small RNAs in tomato (A), X-axis, length of sRNAs distribution; Y-axis, corresponding percentage of raw reads; The distribution of member number for each evolutionary known miRNA families in tomato (B), X-axis, name of the miRNAs family; Y-axis, representing miRNA numbers**.

For the length distribution analysis, 21–24 nt long RNA reads found significantly abundant throughout the sRNA libraries which are a typical sRNA distribution pattern of high-throughput RNA sequencing. However, 24 nt long sequences showed a remarkably higher abundance only in the shoot tissues (between control and grafted shoot) and comprised approximately 40 and 37% of total, which is four times and two times higher than their corresponding fruit tissues. However, grafted fruit has represented higher abundance (18%) in 24 nt long sequences than the control one (10%). This is an opposite picture of shoot tissues where the control tomato shoot exhibited 3% higher than the grafted shoot. This scenario suggested that 24 nt long sRNAs has a remarkable abundance in shoot tissues while fruit sRNAs are steadily distributed from 21 to 24 nt long sequences. Even though, 24 nt long sRNAs were found the most abundant class across the libraries (Figure 3).

### Identification of evolutionary known miRNAs

To analyze and predict the evolutionary known miRNAs, our sRNA libraries were BLASTN searched against the plant known mature miRNAs deposited in miRBase (v20, June 2013). A total of 68 evolutionary known miRNAs have been identified that have orthologs in other plant species and widely distributed over 37 diversified miRNA families (Figure 3). Between 37 miRNA families, SSG represented all the miRNA families followed by SSC and SFG, each of these represented 34 evolutionary known families. The number of miRNA family found less dramatically in SFC library, which represented only 25. The highest number of known miRNAs was expressed in SSG (68) followed by SSC (65) and SFG (62) and the lowest (44) was expressed in SFC. The results have identified four known miRNAs (miR171c, miR171d, miR530, and miR5302) that showed only shoot specific expression among the sRNA libraries and no known sequences identified as fruit tissues specific (Table 2 and details in Table S2).

**Table 2:** **Summarized information of miRNAs in sRNA libraries of tomato**.

**Type**	**Total**	**sRNA libraries**			
		**SFC**	**SFG**	**SSC**	**SSG**
Known mature miRNAs	68	44	62	65	68
Known miRNA family	37	25	34	34	37
Significantly DE known miRNAs	44				
Novel mature miRNAs	168				
miRNA::miRNA^*^ duplex	98				
Significantly DE novel miRNAs	162				
DE miRNAs with targets	70				
Target number of DE miRNAs	6255				

DE, differentially expressed; miRNA::miRNA^*^, the sequences that can form miRNA::miRNA^*^ with miRNA variants.

The member of evolutionary known miRNA families was further analyzed and distributed in Figure 3, where the majority of families (23 of 37) contained single members. Micro RNA families MIR156, MIR172, and MIR5303 contained highest five members while 11 families viz, MIR162, MIR166, MIR167, MIR168, MIR171, MIR1919, MIR319, MIR398, MIR482, MIR6024, and MIR7997 contained several members (2–4).

Abundance of and expressions of plant miRNAs always vary on spatio-temporal differences (Wang et al., 2012). Results have shown that the abundance of miRNA families like MIR159, MIR166, MIR6022, MIR162, MIR482, and some other were consistently higher throughout the libraries. But SFC has shown comparatively lower abundance for all the miRNA families. At the same time, MIR397, MIR5302, MIR8007 has infrequently sequenced. The possible reason is these miRNAs may be expressed at the lower level under certain condition and in specific tissues. This inconsistent sequencing frequency might be suggested that the conserved miRNAs are well expressed than non-conserved miRNAs in plant tissues; moreover, they may have some distinct physiological roles with diversified regulatory effects in grafted tomato. For example, miR159 is a highly conserved miRNA which controls the expression of some MYB transcription factors and regulates germination, vegetative growth, flowering time, anther development and seed shape (Palatnik et al., 2007). Conversely, some other miRNAs, such as, miR530, miR8007, miR1916, and miR8021 were found at a low-level pattern of expression. This scenario indicates that they have a narrow range of specificity to plants. These miRNAs have less conserved homologs, and their functions are also not clear yet (Liu et al., 2013).

### Prediction of potentially novel miRNAs and nucleotide bias

Another most important feature of deep sequencing is the ability to detect novel miRNAs from sRNA transcriptome. Based on the available tomato genome sequence, we have detected flanking sequences of the grafted tomato candidate miRNAs. Potential novel miRNAs were identified through the exploration of secondary precursor structures, Dicer cleavage sites and by measuring the (MFE). Custom scripts were used to obtain the counts of predicted miRNAs as well as to detect the first nucleotide bias on the first position with certain length along with each position. According to these criteria, a total of 168 novel mature miRNA sequences were identified. Most of the novel miRNA candidates (166) were expressed in shoot tissues (SSC and SSG library) followed by SFG (162). Surprisingly, SFC expressed the lowest (101), which almost half of its corresponding grafted fruit tissues (Table 2). However, the novel miRNAs were found to be consistent between shoot tissues, but it was a tremendous enlargement in grafted fruit compared to its control. This big change at transcription level suggests that the regulatory reaction of miRNA sequences occurred in tomato fruits after grafting on goji and this inflation of transcriptions and miRNAs might be because of the pulling up of miRNAs from root stock to scion.

**Figure 4:**
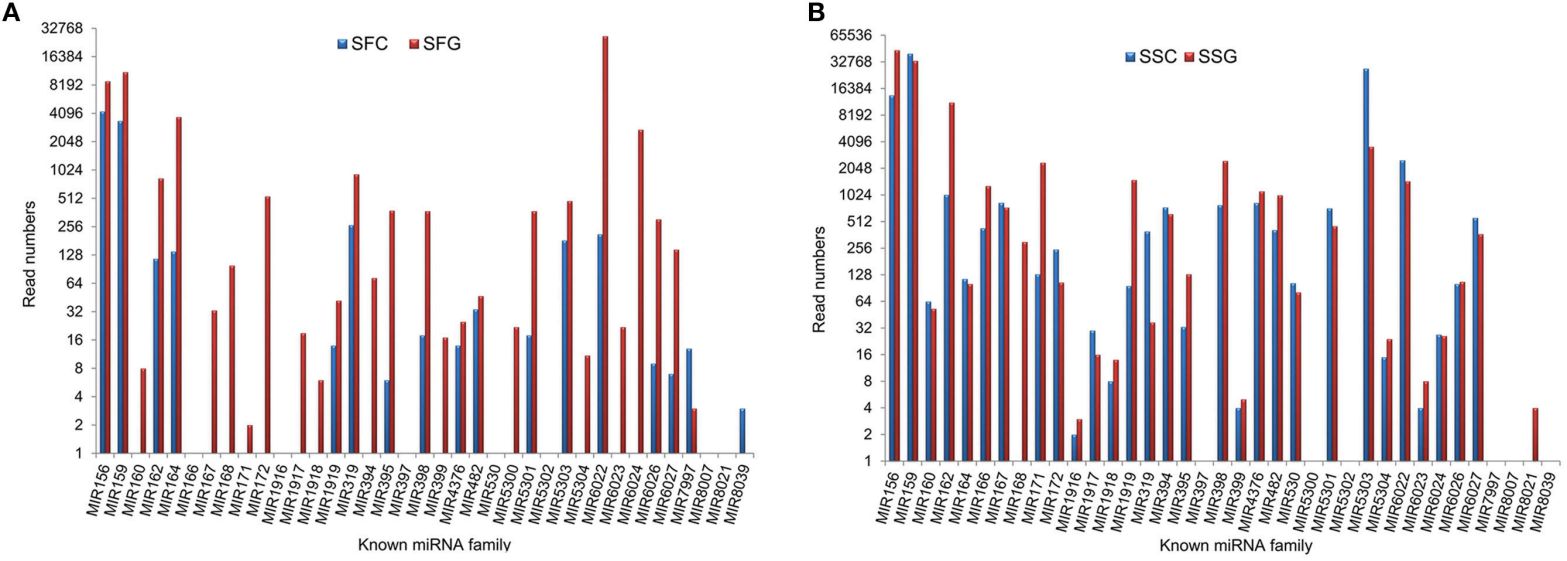
**Expression level (number of raw sequence reads) of evolutionary known miRNAs in each library in tomato**. X-axis, name of the miRNAs family; Y-axis, representing sRNA reads. **(A)** SFC and SFG libraries. SFC, Solanaceae fruit control; SFG, Solanaceae fruit graft. **(B)** SSC and SSG libraries. SSC, Solanaceae shoot control; SSG, Solanaceae shoot graft.

Predicted novel miRNAs found with a low abundance which supports the notion that the non-conserved miRNAs are often expressed at a lower level than the conserved ones (Figure 4). The lowly expressed miRNAs might suggest their specific roles under various habitats, developmental phases or in specific tissues, and their regulation nature remains to be investigated (Table 3).

**Table 3:** **Summary of differentially expressed miRNAs among the sRNA libraries in tomato**.

**miRNA type**	**Total number**	**Category**	**sRNA libraries**	
			**SFG vs. SFC**	**SSG vs. SSC**
Known	68	Differentially expressed miRNAs	64	66
		Significantly differentially expressed	33 (100%)	11 (100%)
		Up-regulated	26 (78.78%)	8 (72.72%)
		Down-regulated	7 (21.21%)	3 (27.27%)
		With fold change ≥ 3	33 (100%)	2 (18.18%)
Novel	168	Differentially expressed miRNAs	160	151
		Significantly differentially expressed	130 (100%)	32 (100%)
		Up-regulated	97 (74.61%)	20 (62.5%)
		Down-regulated	33 (25.38%)	12 (37.5%)
		With fold change ≥ 3	100 (76.92%)	6 (18.75%)

Data of the novel candidate miRNAs were summarized with the nucleotide bias at the first position of all sRNA sequences of certain lengths. This summary could often be helpful to assess the prediction accuracy according to the base bias of known miRNAs. From the libraries, it was observed that the majority candidates started with a 5′ U (uracil) and with a length of 21 nt (Figure S2).

### Differentially expressed known and potentially novel miRNAs between grafted and control tomato

MiRNAs are expressed in a spatio-temporally manner or in the response of environmental stimuli. So their biogenesis is highly regulated. The miRNAs are differentially expressed between libraries and provide a clue at the molecular events related to grafting. Thus, they make a correlation between rootstock and scion. Firstly, the read density measurement (transcripts per million-TPM) was normalized followed by *P* < 0.01 and the absolute value of log2 ratio fold-change > 1.0 were maintained as the threshold for the statistical significance level of miRNAs.

From the Table 4, 33 known miRNAs found significantly differentially expressed between grafted and control fruit sRNA libraries and 11 in shoot libraries. A total of 123 miRNAs (26 known and 97 novels) were up-regulated in the grafted fruit and only 28 miRNAs (8 known and 20 novels) were up-regulated in grafted shoot. Moreover, 133 miRNAs (33 known and 100 novel) found with ≥ 3 log2 fold-changes between fruit samples, while only 8 miRNAs (2 known and 6 novel) in shoot samples. This picture suggests that the fruit of tomato is highly responsive by the grafting technique on goji rootstock and there might be a possible exchange of biologically important phytochemicals from root stock to scion. These grafting results imply miRNAomes, in the form of sRNAs transcripts, a very complex issue (Lu C. et al., 2005), though miRNA sequences thought to be explicitly conserved through plant species (Voinnet, 2009).

**Table 4:** **Significantly differentially expressed novel fruit miRNAs with 3 or more fold change**.

**miRNA**	**MS (5′–3′)**	**ML (nt)**	**PL (nt)**	**Read counts**				miRNA^*^	**FC**	**DES**
				**SFC**	**SFG**	**SSC**	**SSG**			
tono1	aauuuaacuuuagagcuuucuuu	23	126	94	16,712	5	14	Yes	5.5	SFG
tono2	aaccuaguugaauguucaaau	21	294	0	936	15	13	Yes	13.69	SFG
tono3	auguaggcucaggcuaugacug	22	284	0	9	21	13	No	6.99	SFG
tono4	caugugccuguuuuccccauca	22	163	0	2	0	0	No	4.82	SFG
tono5	uuacuuuaguuaaggugugucucu	24	246	0	8	28	18	No	6.82	SFG
tono7	uuaaugugacaaaaauuaguucug	24	173	0	2	66	36	No	4.82	SFG
tono9	gggaaaaagaucugaaauauauu	23	296	0	3	12	6	No	5.4	SFG
tono13	uuugccaugucugaaaucauc	21	85	0	7	9	11	No	6.63	SFG
tono14	aaugauuuaauagaaaaaugacgu	24	242	0	7	32	17	Yes	6.63	SFG
tono16	augcacaaguaccucguagacuau	24	294	0	57	4	2	Yes	9.65	SFG
tono18	ucgacugagucugugugacuug	22	148	0	90	41	116	No	10.31	SFG
tono24	ucggaccaggcuucauuccccc	22	136	1	79	121	86	Yes	4.33	SFG
tono25	caauaaagcugugggaagaua	21	106	9	126	15	1	Yes	−3.78	SSG
tono26	aagugugucuuugaaauuucgauc	24	248	0	12	22	16	Yes	7.4	SFG
tono27	uugagccgcgucaauaucucu	21	85	1	180	10	4	Yes	5.52	SFG
tono30	aagaaaauaaagacuucacagacu	24	56	0	18	77	58	Yes	7.99	SFG
tono34	agggugguggauaagauuuuagga	24	129	0	2	11	11	Yes	4.82	SFG
tono35	aguucuuguagggugagacaac	22	83	0	44	4	11	Yes	9.28	SFG
tono36	cugaaguguuuggggaaacuc	21	69	0	35	3	16	Yes	8.95	SFG
tono37	uaugcuuggguguaugauaugugg	24	290	19	2	6	6	Yes	−5.29	SFG
tono38	auuucucuggugcuuacucaac	22	119	0	23	16	22	Yes	8.34	SFG
tono40	guucccuugaccgcuucauu	20	241	0	7	1	9	Yes	6.63 and 3.29	SFG and SSG
tono41	cgccaaaggagagcugcccug	21	64	0	35	1	9	Yes	8.95 and 3.29	SFG and SSG
tono42	uuuguccuaaaacuaugcgua	21	229	0	15	18	15	Yes	7.73	SFG
tono44	ucgauaaaccucugcauccagc	22	82	0	4	18	7	Yes	5.82	SFG
tono45	ugccaaaggagagcugcccug	21	66	0	13	1	4	Yes	7.52	SFG
tono48	uggagaagcagggcacgugcaa	22	94	0	20	0	1	Yes	8.14	SFG
tono54	aagugugucucugagauuuugggc	24	245	469	148	622	357	Yes	−3.63	SFG
tono55	auagucgaggugugcauaagcugg	24	238	886	236	1210	1054	Yes	−3.87	SFG
tono56	auaagugugucucugagauuucgg	24	237	0	2	1	0	Yes	4.82	SFG
tono57	uuguguauugaagaguguauuacu	24	263	0	9	11	4	No	6.99	SFG
tono62	aaaaguacgacggaagguaucugu	24	236	0	9	58	31	No	6.99	SFG
tono64	auuuauguccuuuaacuuugagug	24	219	0	6	41	17	No	6.4	SFG
tono65	aggucauaguugucaacugaaguc	24	253	0	6	9	5	No	6.4	SFG
tono67	aagugugucucugaaauuucaauc	24	245	52	17	62	35	No	−3.58	SFG
tono69	aaagugagacgaacaaauugaauc	24	62	3	550	633	503	Yes	5.54	SFG
tono70	uuggacugaagggagcuccua	21	176	0	6	36	22	No	6.4	SFG
tono71	auuuaguacacuuuuugaauu	21	80	0	7	36	36	Yes	6.63	SFG
tono73	auaacugugcauuuuaacuugacu	24	289	0	3	29	15	No	5.4	SFG
tono75	uuauuauaguauaagugugucucu	24	222	0	23	58	32	Yes	8.34	SFG
tono76	auucauguaaaacuuuauagacgu	24	149	0	16	2	5	No	7.82	SFG
tono79	cagcugacgacucguugauucu	22	86	0	2	567	918	Yes	4.82	SFG
tono80	auguaacuucgaacuaucguaaau	24	286	0	9	54	36	No	6.99	SFG
tono81	ucaaccuccgacgggcuucgug	22	58	0	46	89	80	No	9.34	SFG
tono82	uggaagggagaauauccaga	20	83	1	111	7	8	No	4.82	SFG
tono83	uuugaucuguaucucuaugac	21	76	194	89	72	96	Yes	−3.09	SFG
tono84	caucgugccggcgacgca	18	73	3	1	0	1	No	−3.55	SFG
tono85	ugucuuugggauuucgaucaua	22	176	0	16	3	19	Yes	7.82	SFG
tono86	agagaacaguggcugagacgg	21	269	410	21	0	0	No	−6.25	SFG
tono91	guuaaggcgugucucugaauuugg	24	94	518	12	5	9	No	−7.40	SFG
tono92	uuaucuggaguuacaaguuga	21	109	0	3	13	9	No	5.40	SFG
tono93	acucucacucgccuuucuca	20	246	13	0	0	0	No	−9.46	SFG
tono94	acucaaauccgagaucucugguua	24	93	4	284	1111	1035	Yes	4.17	SFG
tono96	uuggcauucuguccaccucc	20	107	0	70	752	618	Yes	9.95	SFG
tono104	acacacucugcauucaauuaaauu	24	138	0	77	171	134	Yes	10.09	SFG
tono110	uauuucugcagcuuuggaauu	22	70	1	241	67	34	Yes	5.94	SFG
tono117	aaaauuggauguaucuagcgaauu	24	137	0	4	14	6	Yes	5.82	SFG
tono119	ggaaucuugaugaugcugcag	21	93	0	2	21	19	Yes	10.09	SFG
tono121	cucucccucaaaggcuucugg	20	69	0	2	13	8	Yes	5.94	SFG
tono122	uuaauugucguuaaauauguauu	23	56	0	8	13	8	No	5.82	SFG
tono123	aauguuguuuggcucgaaguc	21	133	0	11	5	10	Yes	4.82	SFG
tono125	aagcgugucucuaaaauuucgagc	23	107	0	14	38	25	Yes	4.82	SFG
tono128	acagaacuaguauaaacguauu	22	276	0	9	17	57	No	6.82	SFG
tono131	agacacaaguaccucuuagacuau	24	290	0	8	17	8	Yes	7.52	SFG
tono132	acuccucgaucuaugucugaaauu	24	269	0	13	9	7	Yes	4.66	SFG
tono134	uuccaugagacuguuuuugggu	22	263	101	10,015	4521	2164	Yes	5.4	SFG
tono137	agguccuauuacccuucugaacuu	24	178	0	3	15	2	No	5.57	SFG
tono138	cuaugagauaaguucaacgug	21	128	20	3749	2479	2165	Yes	−4.78	SFG
tono140	cucggggcguggaccagc	18	151	239	34	58	52	No	−5.54	SFG
tono142	aaggcgugucucugaaauuucagu	24	200	633	53	55	49	Yes	9.03	SFG
tono143	uuuauguccuuuaacuuugagugu	24	217	0	37	136	98	No	6.99	SFG
tono144	aaggucauaguugucaacugaagu	24	246	0	9	28	17	No	−10.11	SFG
tono149	auuagugugucucugaaauuuugg	24	271	47	11	2	8	No	−4.06	SFG
tono156	auguccucugucauacuuuugaga	24	239	0	10	35	27	No	7.14	SFG
tono160	agcuuuaacaaauuugugccaacc	24	105	0	23	58	36	No	8.34	SFG
tono161	acgucugccugggcgucaugc	21	192	0	21	38	35	No	8.21	SFG
tono162	uuuuaacuugaaaauguagagauu	24	107	0	10	21	40	Yes	7.14	SFG
tono163	auaaaugugaucugaagccaaguu	24	241	0	2	29	15	No	4.82	SFG
tono164	auauuaucguuaaggaguuug	21	200	0	1	13	0	No	−6.86	SSG
tono167	uguaggcucaggcuaugacug	21	282	0	2	17	10	No	4.82	SFG

*MS, mature sequence of novel miRNAs; ML, mature length of novel miRNAs; PL, precursor length; FC, Log2-fold change; DES, differentially expressed sample*.

The comparative analysis of miRNAs expression level, a great variation of transcriptional accumulation was observed between grafted and control tomato fruit. Among the evolutionary known miRNAs, 33 were found highly significant with more than 3 fold change with a large range of abundance from 7.09 to 191,474 TPM. Highest fold-change (10.4) observed in miR172a with up-regulation in SFG. Moreover, miR6022, miR166a-5p, and miR6024 were found as the most abundant families with 191,474.2, 4,180.5, and 2,990.12 transcripts, respectively. Conversely, miR166a-5p, miR6022, and miR7997c found as the most representing miRNA families in SFC library with 41,753.1, 5,917.10, and 3,805.84 transcripts, respectively. However, miR166a-5p found consistently higher representing miRNA in both the grafted and control fruit libraries. On the other hand, in the shoot sRNA libraries (SSG vs. SSC) only two known miRNAs (miR398a-3p and miR8021) were found in a fold change more than 3. In case of novel miRNAs, three (tono23, tono38, and tono39) were found with more than 3 fold-change. The highest fold change was identified in tono23, and it was also down-regulated in SSG and other two were up-regulated in control shoot (SSC) (Table S3).

### Target genes prediction, GO, and KEGG analysis of grafted tomato fruits

Micro RNAs and their corresponding target genes exhibit a high sequence complementarity in plant genomes and thus explore target genes directly and successfully (Jones-Rhoades and Bartel, 2004). In the expression regulation of a target gene mechanism, miRNAs usually get hybridized to the mRNA transcripts of a target gene to promote RNA degradation, inhibit translation or both. For the better understanding of the biological functions of the differentially expressed miRNAs in grafted tomato, GO enrichment and KEGG database were analyzed. A total of 6255 genes were found to be associated in the functional annotation that was targeted by 70 significantly differentially expressed miRNAs in this study and three targets (Solyc07g062840.2, Solyc11g027650.1, and Solyc03g121000.2) were complementary to three novel miRNAs (tono50, tono95, and tono97) (Table S4). The rest of the targets were found as the complementary sequence to the known miRNAs. The potential targets of the most miRNAs were varied with a span from few to several hundreds. However, three miRNAs named tono97, miR167a and miR298b-3p have targeted the least number, one in each, while the highest number of genes (981) were targeted by miR5303. Noteworthy, many miRNAs did not match with any target at all; suggesting that this might be due to the absence of targets in the reference genome and/or low expression of these miRNAs in the sRNA libraries, nonetheless, indicates the scope of their functional characterization in tomato grafting.

**Figure 5:**
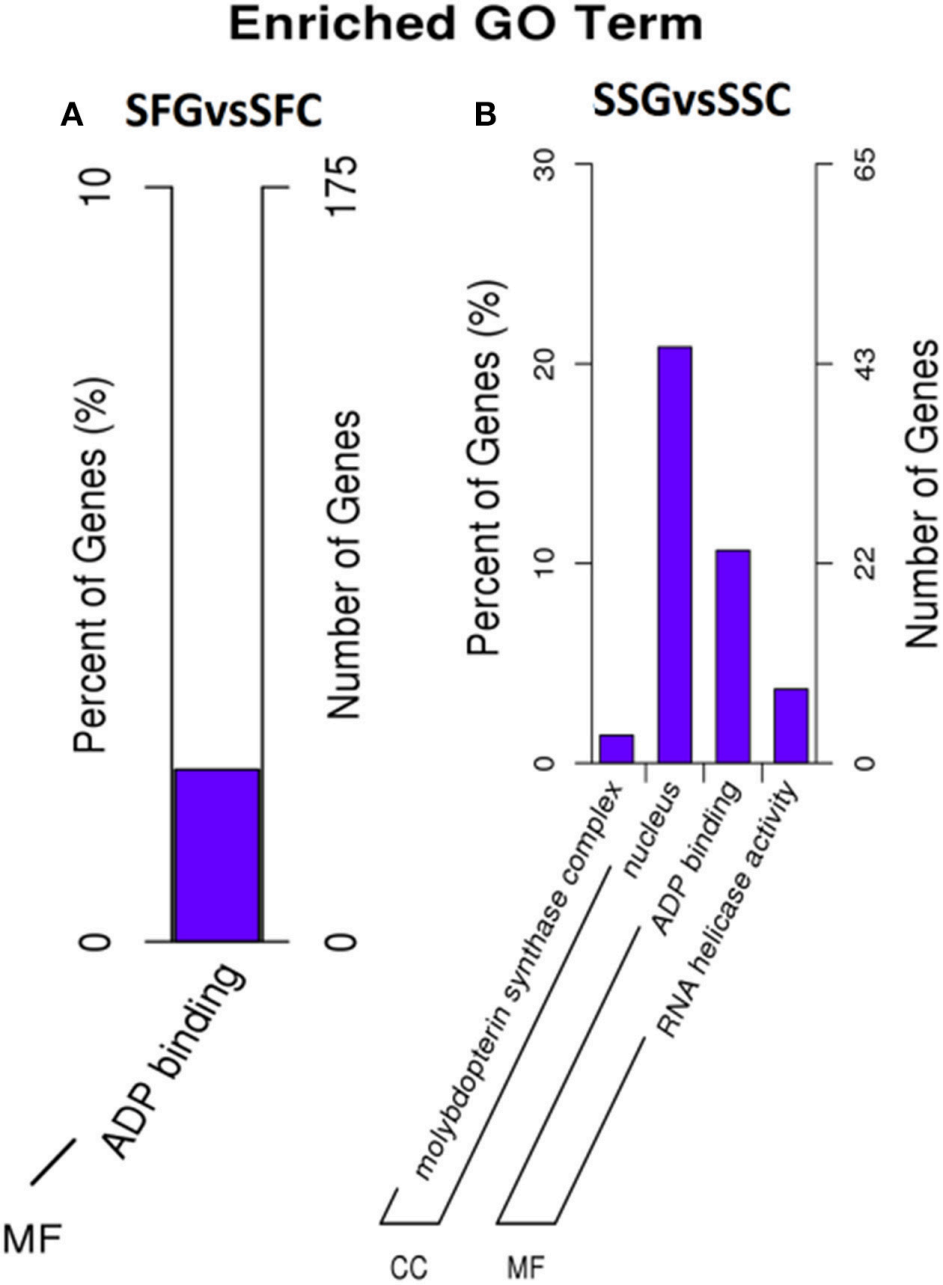
**Gene ontology terms and numbers of predicted target genes for differentially expressed miRNAs from SFG vs. SFC (A) and SSG vs. SSC (B)**. SFG, Solanaceae fruit graft; SFC, Solanaceae fruit control; SSG, Solanaceae shoot graft; SSC, Solanaceae shoot control. CC, Cellular Component; and MF, Molecular Function. Right hand side scale, targeted gene numbers corresponding to the GO terms; left hand side scale, percent of targeted gene numbers corresponding to the GO terms.

GO and KEGG databases are very important for the functional annotation of gene enrichment analysis of the potential targets of significantly differentially expressed miRNAS. A total of 163 miRNAs (33 known and 130 novels) in fruit sRNA libraries (SFG vs. SFC) and 43 (11 known and 32 novels) miRNAs shoot (SSG vs. SSC) libraries were considered for the functional annotation of the targets following some previous reports (Kanehisa and Goto, 2000; Mao et al., 2005; Young et al., 2010). There are three components of GO, such as BP (biological process), CC (cellular component), and MF (molecular function). Over-represented *P*-values followed by corrected *P*-values were employed to identify the involved enriched gene categories in GO enrichment analysis. KEGG analysis was assigned for the elucidation and better understanding of different biological pathways. However, GO annotation was assigned to the all putative targets as the basis of GO components. In SFG vs. SFC libraries, only the “molecular function” was found as the enriched GO term. Among these “ADP binding regulators,” targets were identified as highly significant and over-representing, which is 2.28% of the total MF related targets and regulated by 40 genes (Figure 5 and Table S5). However, SSG vs. SSC libraries showed diversified regulatory activities in cellular component and also in molecular functions. A total of 45 genes were involved in regulation at the nucleus followed by ADP binding activities and 23 genes are involved, moreover, molybdopterin synthase complex and RNA helicase activity were as well found significantly enriched, associated with 3 and 8 genes, respectively.

**Figure 6:**
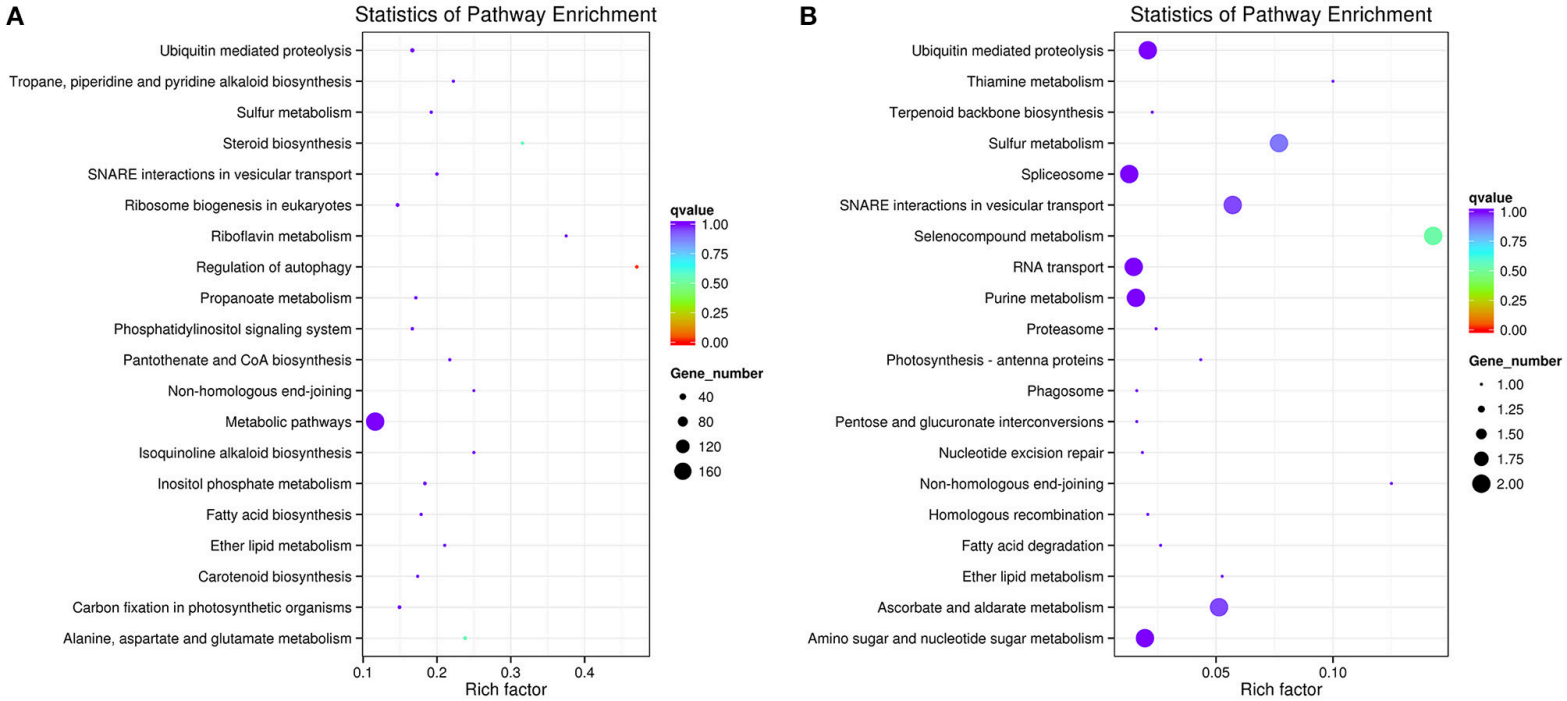
**KEGG analysis with 20 most enriched pathways from SFG vs. SFC (A), and SSG vs. SSC (B)**. Coloring of *q*-values indicates the significance of rich factor; circle indicates target genes involved and size is proportional to the gene number.

For the elucidation of important biological pathways' interaction and the target genes of differentially expressed miRNAs, we further have followed KEGG pathway enrichment analysis. After analyzing the target genes of miRNAs and the corrected *P*-values, a highly diversified 113 biochemical pathways were found to be enriched in fruit tissues. From these, 20 pathways have been identified on the basis of rich factor analysis; corrected *q*-value and number of involved genes in SFG vs. SFC libraries. A total of 298 target genes of the miRNAs were associated. However, “metabolic pathways” was found as the most significant pathway term in respect of rich factor and was regulated by the highest number of genes 172, followed by the ubiquitin mediated proteolysis (16 genes) and ribosome biogenesis in eukaryotes (11 genes). In SSG vs. SSC libraries, only 29 genes were found to be involved in the top 20 enriched biological pathways. Moreover, the number of involved genes in SSG vs. SSC was not more than two in any pathways. However, the enriched pathways were related to selenocompound metabolism, sulfur metabolism, and some other (Figure 6). Details of the pathway terms, id name, gene name and hyperlink of pathways are enlisted in Table S5.

### Analysis of graft-transmissible miRNAs as a whole miRNA movable molecule

**Figure 7:**
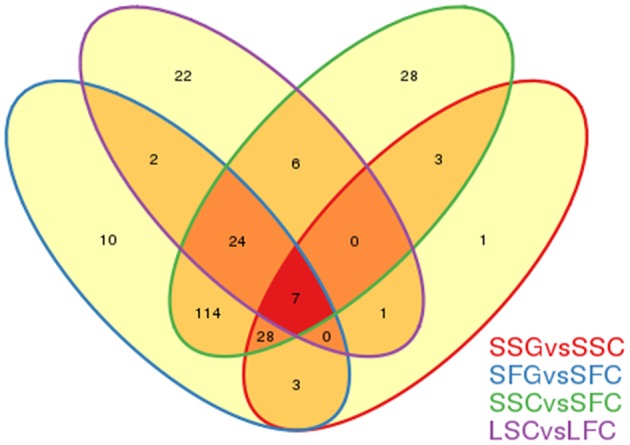
**Common and specific response differential miRNAs in grafted tomato plants and their controls from compared libraries**. SFC, Solanaceae fruit control; SFG, Solanaceae fruit graft; SSC, Solanaceae shoot control; SSG, Solanaceae shoot graft; LSC, goji shoot control; LFC, goji fruit control.

In our previous study on sRNAs characterization of goji control plants (Khaldun et al., 2015), 62 miRNAs found to be significantly differentially expressed in shoot and fruit tissues. We have further analyzed that information with present study (where goji is the rootstock) to identify common and specific significantly differentially expressed miRNAs in both shoot and fruit tissues of grafted tomato plant and their corresponding control plants. Figure 7 representing that only one significantly differentially expressed miRNA (tono154) was specific to SSG vs. SSC combination, indicating that there was no remarkable difference in miRNAs number between control and grafted tomato shoot, whereas 10 miRNAs found that were shown expression only in grafted and control tomato fruit (SFG vs. SFC) and was not found in other combinations. Interesting observation is that SSC vs. SFC showed specific difference in 28 miRNAs and LSC vs. LFC showed in 22, suggesting that numbers of miRNAs and regulating patterns widely variy between shoot and fruit tissues. However, six significantly differentially expressed miRNAs were found common in both shoot and fruit tissues of tomato and *Lycium* (LSC vs. LFC and SSC vs. SFC). Two miRNAs (goji novel28 and miR8007a-5p) were common between shoot and fruit in tomato and *Lycium* (LSC vs. LFC and SFG vs. SFC) whereas three (miR395a, miR6023, and miR8021) were common in both shoot and fruit of tomato (SFG vs. SFC and SSG vs. SSC). However, no miRNAs found common between grafted fruit and shoot of tomato and the fruit and shoot of *Lycium*. After analyzing the RNA reads count, three miRNAs (goji novel28, goji novel9, and miR8007a-5p) found as the transported from rootstock of goji to grafted tomato plant (Table 5). In the qRT-PCR experiment these miRNAs were expressed in the control tomato samples in a very low read counts. So, the sequencing data for these three miRNAs somehow showed expression deviation in the qRT-PCR results. But, one thing is clear these miRNAs shown strong expression in goji samples whereas they have shown a very weak level of expression. However, further experiment can identify additional information for these miRNAs as a whole miRNA movement from rootstock to the scion.

**Table 5:** **Expression pattern of the graft-transmissible miRNAs in the small RNA libraries**.

**miRNAs**	**Read counts (TPM)**						**Significantly DE shown between the samples**
	**Results in tomato sRNA libraries**				**Results in gouqi sRNA libraries**		
	**SFC**	**SFG**	**SSC**	**SSG**	**LSC**	**LFC**	
Gouqi novel28	0.00	5.44	0.00	0.00	106.96	0.00	SFG vs. SFC, LSC vs. LFC
stu-miR8007a-5p	0.00	5.44	0.00	4.86	35.65	0.00	SFG vs. SFC, LSC vs. LFC
Gouqi novel9	0.00	5.44	0.00	0.00	14,581.63	5889.48	SFG vs. SFC

### Expression validation of sequencing data with qRT-PCR and RLM 5′-race

**Figure 8:**
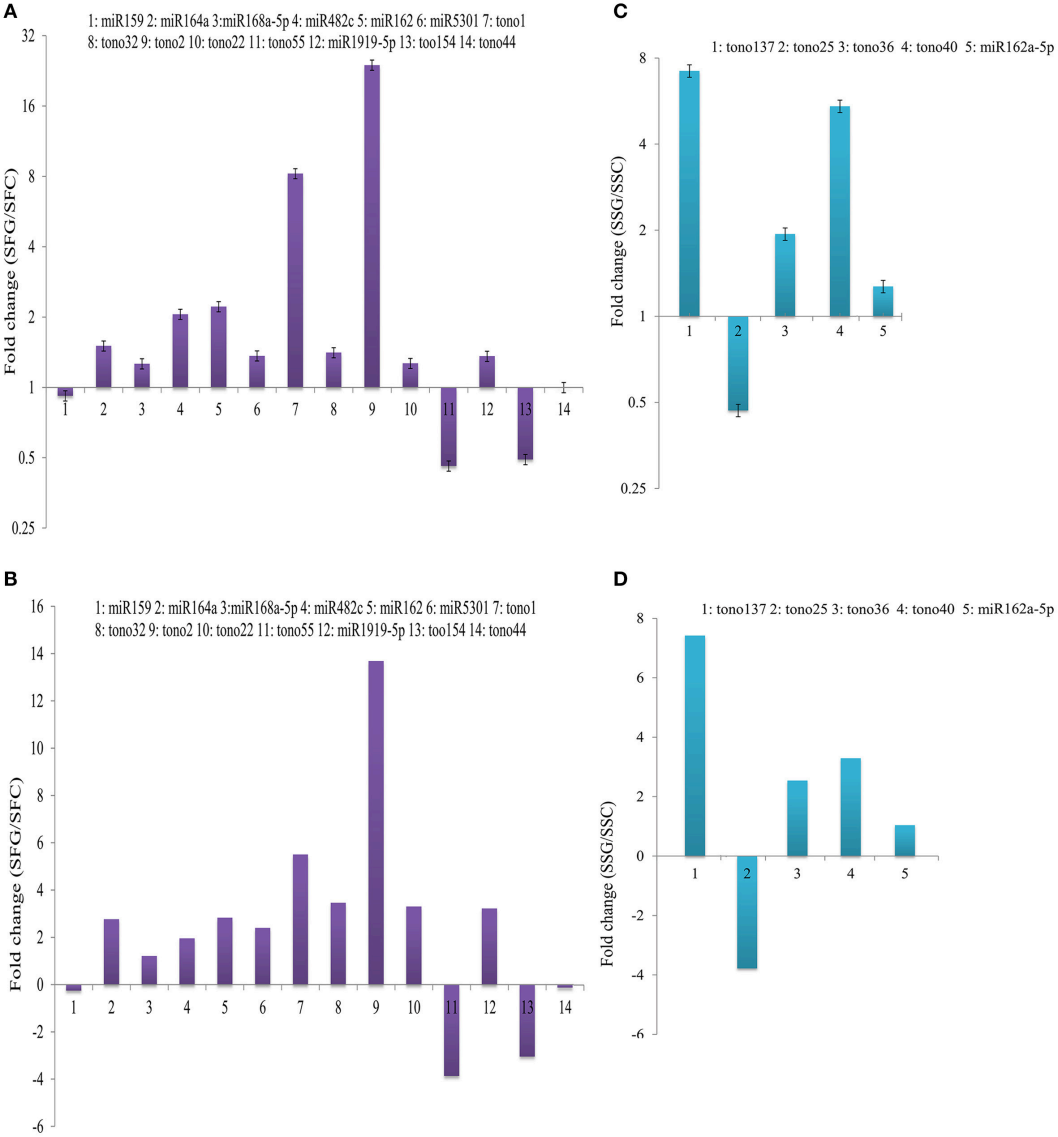
**Expression ratios (grafted fruit/control fruit) of miRNAs in (A) qRT-PCR and (B) sequencing results; and (grafted shoot/control shoot) ratios in (C) qRT-PCR and (D) sequencing results**. X-axis, name of the miRNAs that were selected for qRT-PCR; column above the X-axis, miRNAs that were up-regulated in the grafted fruit and shoot; column below the X-axis, miRNAs that were up-regulated in the grafted and control shoot.

Quantitative RT-PCR was used to validate the sequencing data experimentally. For this, 19 miRNAs were selected, 14 of which were selected from the differentially expressed between grafted and control fruits and 5 between grafted and control shoot sRNA libraries after determining the appropriate reference genes (Figure 8). The C_T_ values and RQ values of these miRNAs were obtained, and 17 miRNAs had a similar expression pattern in the grafted and control tomato samples compared with the sequencing results. However, miR1919-5p and tono2 showed acceptable inconsistency in the expression profile between the sequencing data and RT–qPCR. The possible reasons for this inconsistency might be the difference to the amplification performance of primers or other unknown reasons. The primers that were used for this study are listed in supporting Table S6. Additionally, six important targets were validated by modified RLM 5′-RACE with their miRNA sequencing numbers and cleavage sites (Figure S3).

## Discussion

An accurate mechanism of the plant development and response to the environmental stimuli could be achieved by the gene regulation through sequence-specific interaction between miRNAs and their targets. Therefore, a holistic effort is required for the unraveling of the genetic mechanisms, especially addressing the roles of miRNAs in the grafting system. Discovery of whole sets of miRNAs and their targets may play a centric role in elucidation of complicated miRNA-mediated regulatory network for the graft system that controls the plant growth and development, and other physiological and biological processes. Undoubtedly, the advent of deep sequencing platforms have significantly widened the ability of miRNA exploration and in return provided a new horizon to identify the conserved, non-conserved, lowly expressed as well as species-specific miRNAs in a large scale. By deep sequencing, not only the conserved miRNAs but also the novel miRNAs were identified. To the best of authors' knowledge, this is the first report on high-throughput RNA sequencing of grafting between two distantly related medicinally important herb and shrub. Moreover, the differential expression was observed for the long-distance translocation of miRNAs over a long period of time (from young shoot to mature fruit). Quality control data and the size distribution have confirmed the reliability of throughput sequencing data, which was further validated by the qRT-PCR and the modified 5′RLM-RACE experiments.

### miRNAome in shoot and fruit tissues of grafted and control tomato

High-throughput sequencing is rapidly replacing the conventional Sanger sequencing platforms though the most conserved plant miRNAs have been identified through traditional approaches. By now, the most plant miRNAs have been discovered in *Arabidopsis*, rice and poplar through the traditional methods (Lu S. F. et al., 2005). Some recent studies have been reported that non-conserved, and species-specific miRNAs are normally found at a lower abundance compare to conserved miRNAs, which is literally hard to reveal efficiently and cost-effectively (Moxon et al., 2008). However, deep sequencing has been successfully applied both in model (Lu et al., 2006; Rajagopalan et al., 2006; Fahlgren et al., 2007) and non-model plants (Morin et al., 2008; Qiu et al., 2009) addressed as blessings for miRNAs identification study (Song et al., 2010).

Populations and the distribution patterns are distinguishing features of sRNA libraries. Composition of sRNAs often indicates the roles of different type of sRNAs in specific tissue types, or even particular species. Most of the miRNAs with known functions are 20–24 nt long. In our four libraries, both of the shoot libraries (SSC and SSG) comprised of the 24 nt sRNAs with a remarkably higher frequency (Figure 2). On the other hand, both the fruit libraries (SFC and SFG) are comprised of 21 nt long sRNAs. This is a good indication that 24 nt long sRNAs play more roles in shoot or vegetative tissues of tomato, on the contrary; 21 nt sRNAs have important roles in fruit tissues. In some plant species like grapevine, *Pinus cordata* and *Populus balsamifera*, 21 nt has substantially more than 24 nt sRNAs (Barakat et al., 2007; Morin et al., 2008; Pantaleo et al., 2010). The opposite scenario also reported in some other studies where 24 nt class shown higher-frequency (Rajagopalan et al., 2006; Fahlgren et al., 2007; Szittya et al., 2008). The high frequency of 24 nt sequence may reflect the complexity of tissue samples since 24 nt sequences are considered as siRNAs. siRNAs are known to be involved in heterochromatin modification, specially genomes with content of repeat sequences (Herr, 2005). Moreover, a previous study was reported that 24 nt sRNAs was predominantly comprised of repeat and transposons (Lippman and Martienssen, 2004).

Until date, nearly 22 miRNA families were reported as frequently distributed at least in 20 plant species thus considered as highly conserved and found in dicots and monocots (Sunkar and Jagadeeswaran, 2008). Well-conserved miRNA families have retained homologous target interactions and performed analogous molecular functions in the plant kingdom in evolutionary timescales (Axtell et al., 2007). Gene duplication event is thought to generate plant MIR genes, which then evolved by random mutations into short, imperfectly paired hairpins (Allen et al., 2004; Axtell, 2008). Those miRNAs are mostly non-conserved and are believed to be evolutionarily young and mainly represented by a single copy MIR gene (Jones-Rhoades et al., 2006). Recent studies reported on many non-conserved miRNAs in several species. These non-conserved miRNAs are found among distinct species and in different phylogenetic families. We also have found several non-conserved miRNAs in our study, although they were found in a very low level of expression. Nevertheless, this situation does not rule out that they also could be expressed highly in other tissues. Apart from conserved miRNAs, the abundance of novel miRNAs in our study found at a lower level suggesting their involvement more specific processes to grafted tomato. This also supports the results of previous reports (Liu et al., 2013) where species-specific miRNAs were believed to be newly evolved and lowly expressed than the conserved ones. Therefore, further functional characterization of these novel miRNAs may reveal interesting and useful information about their role in signal transduction and development in grafted tomato on goji. Micro RNAs usually undergo some minimum annotating criteria regarding biogenesis and expression and convincingly detected by either of the methods like northern blotting, qRT-PCR or sequencing analysis (Song et al., 2010). Additionally sequence detection or cloning data of miRNA^*^ are required as a biogenesis proof since they are complementary to mature miRNA sequences (Jones-Rhoades et al., 2006; Meyers et al., 2008). Among the 168 novel miRNAs, 98 were found with their complementary miRNA^*^ sequences (Table 2).

### Target annotation of differentially expressed miRNAs

Some miRNAs such as, miR156, miR162, miR164, miR166, miR172, miR397, and miR398 were reported to be highly conserved in tomato fruit and developmental stages (Zuo et al., 2012; Karlova et al., 2013). In our present study, homologs of miR162, miR164, miR166, and miR397 were identified as significantly differentially expressed between grafted and control tomato fruits but not in the shoot tissues (Table S7). All these miRNAs found to be up-regulated in grafted fruit. Surprisingly, these miRNAs were detected as significantly differentially expressed between goji fruit and shoot, where they were up-regulated in fruit (Khaldun et al., 2015). This accumulation difference of these miRNAs in grafted tomato fruit may be due to the long-distance movement after grafting. MiR398 exhibited an interesting expression pattern where it was found as significantly expressed in grafted tomato fruit though it was down regulated in control tomato samples (Table S8). Moreover, it has found significantly differentially expressed in goji fruit, suggested this induction probably due to grafting method. This phenomenon could be a proof of long distance movement of miRNAs from rootstock to scion, though their expression was remarkably low in grafted tomato fruit. These results convincingly implicated that grafting could indeed affect miRNAs expression in scion.

Most of the cases in plants, targets of miRNA family mostly belong to the same gene family (Karlova et al., 2013). Additionally, the perfect sequence complementarity between miRNA and targets conferred the target prediction process straightforward and efficient (Jones-Rhoades and Bartel, 2004). Number of target genes of a single miRNA has counted from one to more than several hundred in our study. Mostly, a single miRNA can work on many targets; similarly, a single gene could be regulated by many miRNAs, making the functional annotation extremely complex. Thus, from high-throughput sequencing method enriched GO annotation and KEGG pathway analyses are efficiently used for the functional annotation of miRNAs with their targets since it is not possible “single gene functional characterization” from such huge data. “Metabolic pathways” was identified as the most enriched pathway between grafted and control fruit. Most of the known miRNAs were also involved in this regulatory mechanism. “Amino sugar and nucleotide sugar metabolism” has identified as the most enriched pathway between grafted shoot and control shoot, where only two genes and two miRNAs involved. So, these genes and miRNAs could be a good source of biological pathway regulation process and should be paid special attention in the future study. The targeted genes of graft-mediated miRNAs encoded proteins of diverse functions such as transcription factors, mostly, and others were involved in metabolism, signal transduction, growth, development and other biological processes (Table S5). Hormones, nucleic acids, metabolics, and proteins can act as systemic signals that could provide useful exchange information between rootstock and scion. It is expected for the conserved miRNAs that their targets should also be well-conserved. Most of the conserved miRNAs (such as miR156, miR159, miR160, miR164, miR167, miR171, miR172, miR319, and some others) usually target a range of transcription factors like MYBs, ARFs, SBPs, NACs, AP2-like factors, GRFs, and GRASs, and their miRNAs-mediated regulations are important for plant growth and development and may act in the core gene expression networks (Liu et al., 2013). Molecular information can move bidirectional in grafting system; however, signaling between rootstock-scion and scion-rootstock requires further studies. In comparison with grafted tomato, and control samples of tomato and goji we have found many miRNAs were differentially expressed.

## Conclusions

Present study reported the miRNAs profiling and target annotation in grafted tissues between two important plants *L. chinense* and tomato. This report, to our knowledge, is the first elucidation of miRNAs in grafting between distantly related two plants. High-throughput sequencing has employed, and 68 evolutionary known miRNAs identified belonging to 37 diversified families. Additionally, 168 putative novel miRNAs were identified from shoot and fruit tissues of grafted and control tomato. A total of 163 (33 known and 130 novels) miRNAs were explored as significantly differentially expressed between fruit sRNA libraries. Moreover, GO analysis has detected “ADP binding activities,” “molybdopterin synthase complex,” and “RNA helicase activity” as the most enriched terms, and KEGG analysis has identified “metabolic pathways” as the most enriched pathway involving 172 genes in grafted and control fruit tissues. Therefore, this study provided a unique insight of graft-oriented miRNAs and their complex regulatory functions in *Lycium* and tomato, which would be helpful for studying the various biological pathways as well as the miRNA translocation in a distant-grafting system.

## Author contributions

AK and YW conceived and designed the experiments. AK, WH, HL, and SL performed the experiments. AK, WH, and SZ analyzed the data. YW, HL, and SL contributed chemicals/reagents/materials/analysis tools. AK, YW, and WH wrote the paper.

### Conflict of interest statement

The authors declare that the research was conducted in the absence of any commercial or financial relationships that could be construed as a potential conflict of interest.

## Supplementary material

Figure S1 **Flowchart of the small RNAs indentification bioinformatics**.

Figure S2 **First nucleotide bias of novel miRNA candidates in tomato. (A)** miRNA first nucleotide bias in SFC, **(B)** miRNA first nucleotide bias in SFG, **(C)** miRNA first nucleotide bias in SSC, **(D)** miRNA first nucleotide bias in SSG. Number on top of the bars indicates the number of sequences corresponding to the miRNA length; X-axis, length of miRNAs; Y-axis, representing percent of each nucleotide.

Figure S3 **Target validation of known tomato miRNAs**. Arrows showing the 5′-ends of the cleavage products.

Table S1 **The designed primers for modified RLM 5′-RACE and targets annotation in tomato**.

Table S2 **Statistics of evolutionary known and novel miRNAs in grafted and control tomato plants**.

Table S3 **Details of differentially expressed evolutionary known and novel miRNAs in grafted and control tomato plants**.

Table S4 **Differentially expressed miRNAs and their corresponding genes in grafted tomato**.

Table S5 **GO annotation, KEGG enriched pathways and corresponding genes in tomato**.

Table S6 **The primer designed and candidate list of miRNAs for qRT-PCR with differential expression through deep sequencing**.

Table S7 **Significantly up-regulated evolutionary known miRNAs with 2.4-, or more fold-change in grafted tomato**.

Table S8 **Significantly down-regulated evolutionary known miRNAs in grafted tomato**.
