# Supplementary figures and images for: Comparative Profiling of miRNAs and Target Gene Identification in Distant-Grafting between Tomato and Lycium (Goji Berry)
Source: Front Plant Sci. 2016 Oct 18;7:1475. doi: 10.3389/fpls.2016.01475 (PMC5067468; doi:10.3389/fpls.2016.01475)

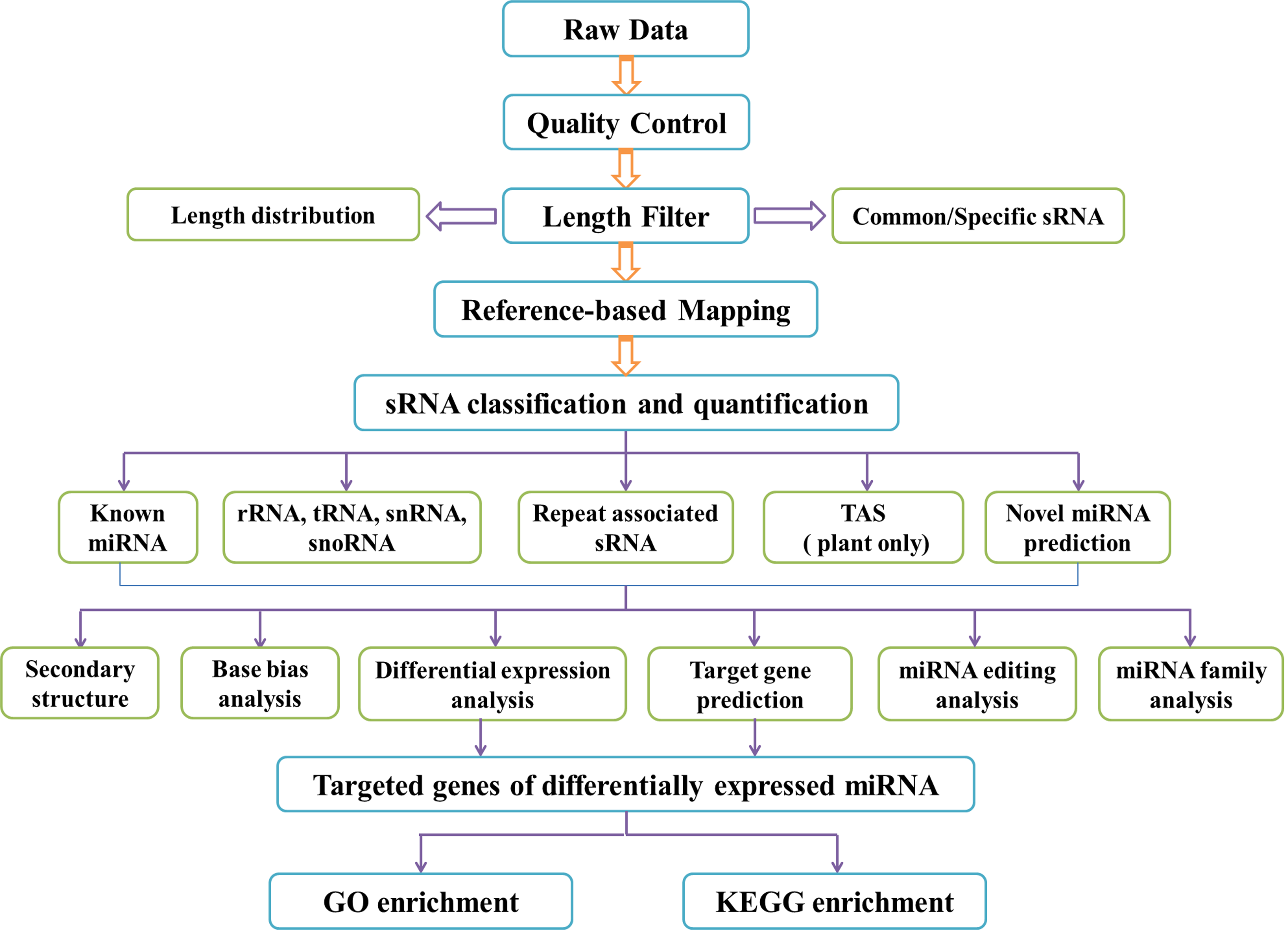

Supplement: Figure S1 — Flowchart of the small RNAs indentification bioinformatics. [file Image1.TIF]

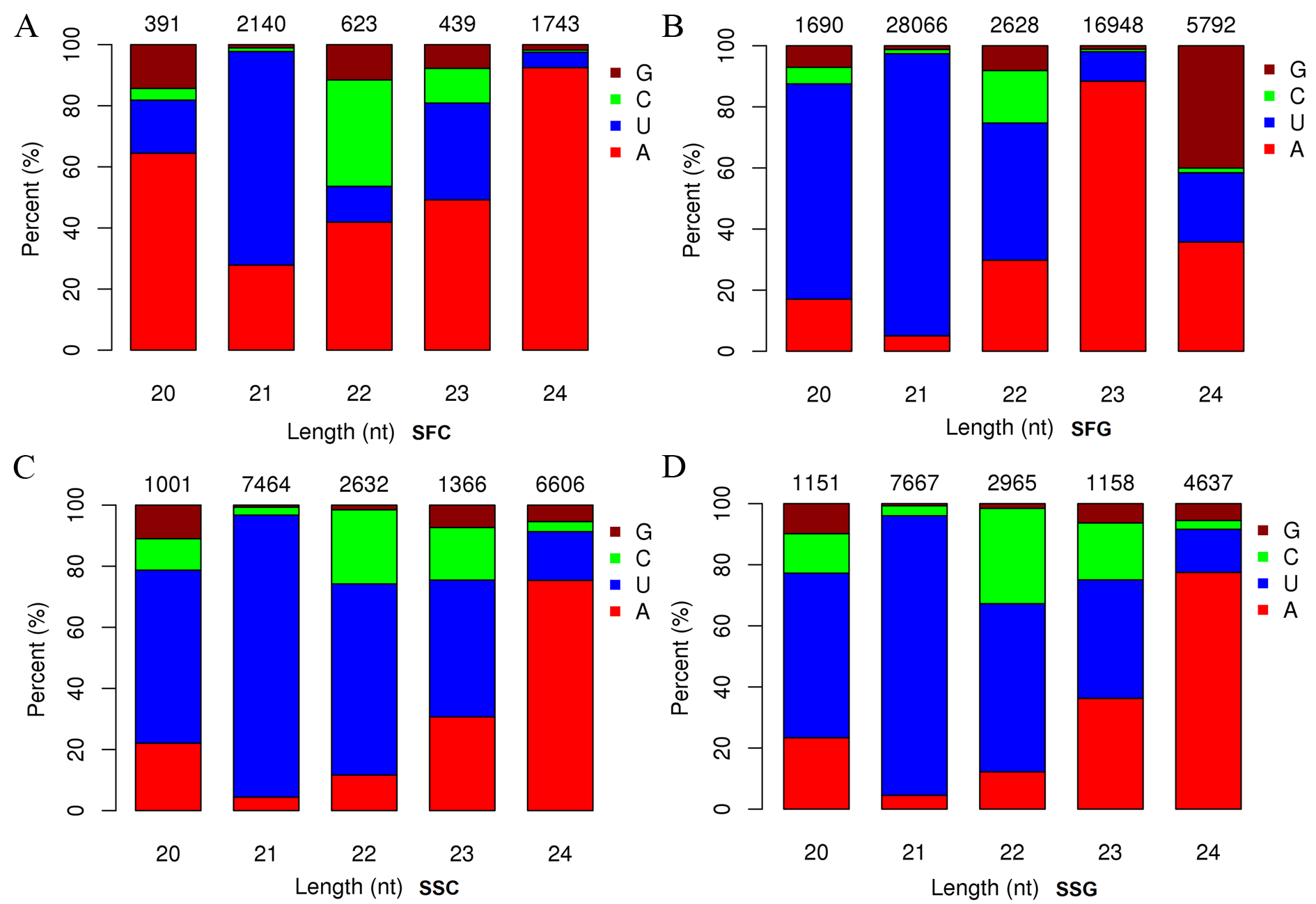

Supplement: Figure S2 — First nucleotide bias of novel miRNA candidates in tomato. (A) miRNA first nucleotide bias in SFC, (B) miRNA first nucleotide bias in SFG, (C) miRNA first nucleotide bias in SSC, (D) miRNA first nucleotide bias in SSG. Number on top of the bars indicates the number of sequences corresponding to the miRNA length; X-axis, length of miRNAs; Y-axis, representing percent of each nucleotide. [file Image2.TIF]

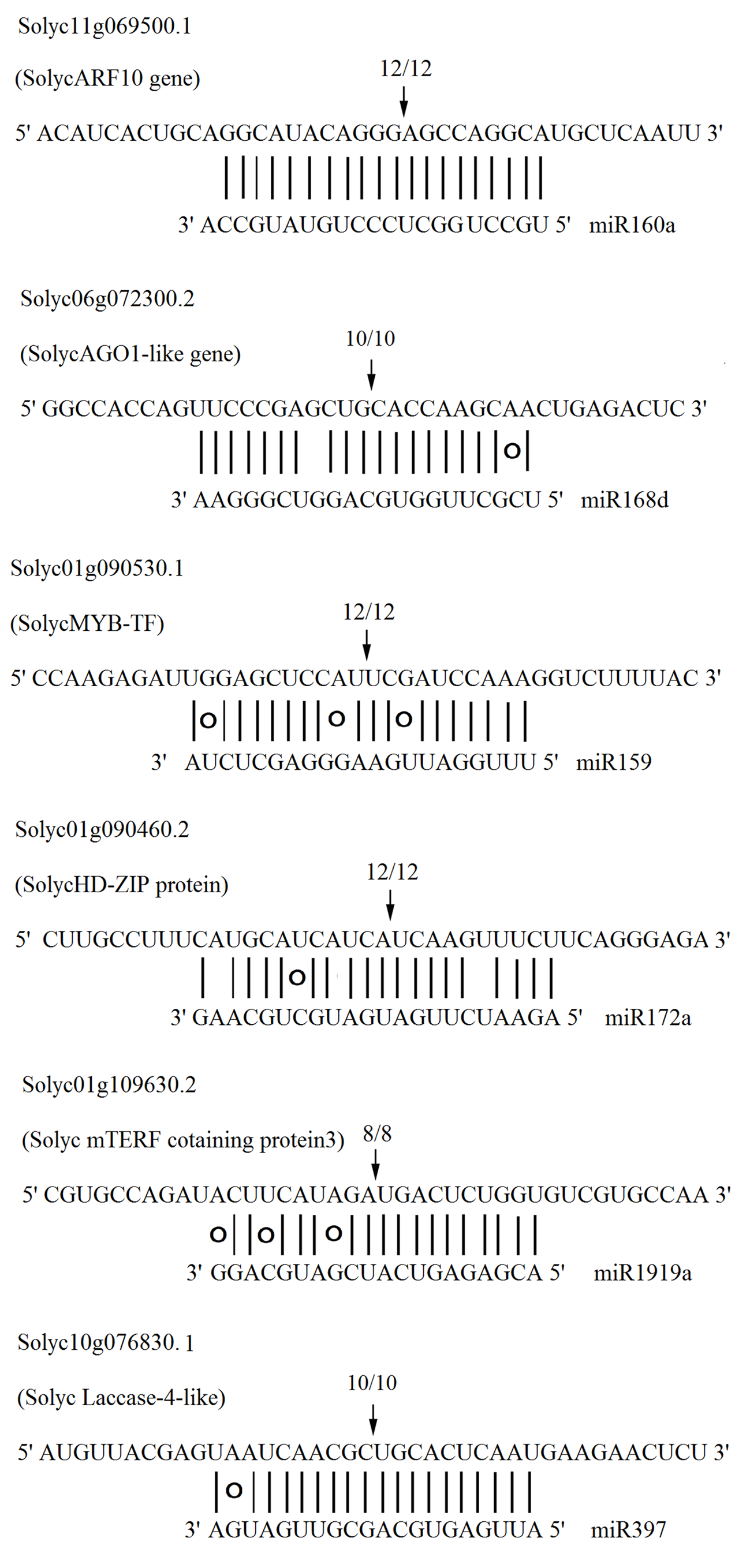

Supplement: Figure S3 — Target validation of known tomato miRNAs. Arrows showing the 5′-ends of the cleavage products. [file Image3.TIF]
