# Supplementary material for: Comparative Profiling of miRNAs and Target Gene Identification in Distant-Grafting between Tomato and Lycium (Goji Berry)
Source: Front Plant Sci. 2016 Oct 18;7:1475. doi: 10.3389/fpls.2016.01475 (PMC5067468; doi:10.3389/fpls.2016.01475)
Supplement: Table S1 — The designed primers for modified RLM 5′-RACE and targets annotation in tomato. [file Table1.DOCX]

**Table S1. The designed primers for modified RLM 5’-RACE and targets annotation in tomato**

| **Target genes** | **Primer sequence (5’-3’)** | **Annotation** |
| --- | --- | --- |
| Solyc11g069500.1: outer primer | TTCAGCAGTTGCGAGACCAGTT | Auxin response factor 10 (ARF 10) |
| Solyc11g069500.1: inner primer | GGGTCAGCATATCTGGTCTTTCAA |  |
| Solyc06g072300.2: outer primer | TGGCAACTCACGCAGAACAATA | Protein argonaute 1A-like |
| Solyc06g072300.2: inner primer | GCAGCCAGCTTGATCACAACTTTA |  |
| Solyc01g090530.1: outer primer | ACACGAAGGAGCAAGAGGGACT | MYB transcription factor |
| Solyc01g090530.1: inner primer | GAGGAAAAGGGAGCGGGAACT |  |
| Solyc01g090460.2: outer primer | ACCTCTGCACATCCAGTGTTTTAG | HD-ZIP protein |
| Solyc01g090460.2: inner primer | GGTTGTTGGAGGGCTCATGTT |  |
| Solyc01g109630.2: outer primer | AGCCTGGGAAATCTGACAAGCATA | mTERF domain-containing protein 3 |
| Solyc01g109630.2: inner primer | AACACTGGCCTGGTAACAAGAATC |  |
| Solyc10g076830.1: outer primer | GTCCCACTCATCGTAGCCAAATTA | Laccase-4-like |
| Solyc10g076830.1: inner primer | AACTCCTAACCCCACGGTGAAA |  |

**Table S6. The primer designed and candidate list of miRNAs for qRT-PCR with differential expression through deep sequencing**

| **Known miRNAs** | | | | | | |
| --- | --- | --- | --- | --- | --- | --- |
| **No** | **Name** | **Mature sequence** | **SL (nt)** | **GrR** | **CtR** | **Primer (5’-3’)** |
| 1 | miR159 | UUUGGAUUGAAGGGAGCUCUA | 21 | 33771 | 40629 | CGGGTTTGGATTGAAGGGAGCTCTA |
| 2 | miR164a | UGGAGAAGCAGGGCACGUGCA | 21 | 3745 | 140 | GGCTGGAGAAGCAGGGCACG |
| 3 | miR168a-5p | UCGCUUGGUGCAGGUCGGGAC | 21 | 438 | 48 | CCCGCCTTGCATCAACTGAAT |
| 4 | miR482c | UCUUGCCAAUACCGCCCAUUCC | 22 | 138 | 9 | TTTCCTATTCCACCCATGCCAA |
| 5 | miR162 | UCGAUAAACCUCUGCAUCCAG | 21 | 2466 | 88 | GCCTCGATAAACCTCTGCATCCAG |
| 6 | miR5301 | UGUGGGUGGGGUGGAAAGAUU | 21 | 374 | 18 | TGTGGGTGGGGTGGAAAGATT |
| 7 | miR162a-5p | GGAGGCAGCGGUUCAUCGAUC | 21 | 182 | 2563 | GGAGGCAGCGGTTCATCG |
| 8 | miR1919-5p | UGUCGCAGAUGACUUUCGCCC | 21 | 159 | 23 | CGCCACGAGAGTCATCTGTGACA |
| **Novel miRNAs** | | | | | | |
| 1 | Tono1 | AAUUUAACUUUAGAGCUUUCUUU | 23 | 16712 | 94 | CGGCGGAATTTAACTTTAGAGCTTTC |
| 2 | Tono2 | AACCUAGUUGAAUGUUCAAAU | 21 | 936 | 0 | CGGCGAACCTAGTTGAATGTTCA |
| 3 | Tono22 | UUGGCUGAGUGAGCAUCACUG | 21 | 12287 | 317 | GGTTGGCTGAGTGAGCATCACTG |
| 4 | Tono25 | CAAUAAAGCUGUGGGAAGAUA | 21 | 1 | 15 | CGTCAATAAAGCTGTGGGAAGATA |
| 5 | Tono32 | UUGGCUGAGUGAGCAUCACGG | 21 | 2316 | 2221 | CGTTGGCTGAGTGAGCATCACG |
| 6 | Tono36 | CUGAAGUGUUUGGGGAAACUC | 21 | 16 | 3 | GGCTGAAGTGTTTGGGGAAACTC |
| 7 | Tono40 | GUUCCCUUGACCGCUUCAUU | 20 | 7 | 0 | GCGGTTCCCTTGACCGCTTCATT |
| 8 | Tono44 | UCGAUAAACCUCUGCAUCCAGC | 22 | 4 | 0 | GCGTCGATAAACCTCTGCATCCAGC |
| 9 | Tono55 | AUAGUCGAGGUGUGCAUAAGCUGG | 24 | 1054 | 1210 | CGATAGTCGAGGTGTGCATAAGCTG |
| 10 | Tono137 | AGGUCCUAUUACCCUUCUGAACUU | 24 | 3 | 0 | CGGAGGTCCTATTACCCTTCTGAAC |
| 11 | Tono154 | AUAAGUGUGACUCUGAAAUUUCGG | 24 | 21 | 10 | CCGCCATAAGTGTGACTCTGAAAT |

**Note:** SL=Sequence length, GrR= Grafted sample reads, CtR= Control sample reads.

**TABLE S7. Significantly up-regulated evolutionary known miRNAs with 2.4- or more fold-change in grafted tomato**

| **miRNA** | **Sequence** | **Target tissue** | **log2- fold change** | ***P*-value** | **Signature** | **Sig-level** | **Key roles** |
| --- | --- | --- | --- | --- | --- | --- | --- |
| miR156h-3p | GCUCACUGCUCUAUCUGUCACC | fruit | 3.82 | 0.04 | true | ** | Tomato fruit ripening  Over expression results in fruit formation and yield |
| miR160a | UGCCUGGCUCCCUGUAUGCCA | fruit | 6.82 | 3.01E-12 | true | ** | Involved auxin response and plant hormone signaling |
| miR162 | UCGAUAAACCUCUGCAUCCAG | fruit | 2.83 | 0 | true | ** | Targets DCL genes in sRNA production |
| miR164a | UGGAGAAGCAGGGCACGUGCA | fruit | 2.77 | 0 | true | ** | Negative regulators of NAC domain containing TF genes |
| miR168d | UCGCUUGGUGCAGGUCGGGAA | fruit | 7.82 | 4.23E-22 | true | ** | Regulate expression of AGO1, which mediates cleavage functions |
| miR171a | UGAUUGAGCCGUGCCAAUAUC | fruit | 3.82 | 0.04 | true | ** | Targets TF genes of SCL protein and, hormone signaling |
| miR171b | UUGAGCCGUGCCAAUAUCACG | fruit | 3.82 | 0.04 | true | ** | Targets TF genes of SCL protein and, hormone signaling |
| miR172a | AGAAUCUUGAUGAUGCUGCAG | fruit | 7.82 | 4.23E-22 | true | ** | Modulates mRNAs during fruit ripening and, hormone signaling |
| miR172a | AGAAUCUUGAUGAUGCUGCAU | fruit | 10.4 | 4.50E-94 | true | ** | Modulates mRNAs during fruit ripening and, hormone signaling |
| miR172c-3p | AGAAUCUUGAUGAUGCUGC | fruit | 4.82 | 0 | true | ** | Modulates mRNAs during fruit ripening and, hormone signaling |
| miR172d-3p | GGAAUCUUGAUGAUGCUGCAG | fruit | 4.82 | 0 | true | ** | Modulates mRNAs during fruit ripening and, hormone signaling |
| miR172j | GGAAUCUUGAUGAUGCUGCAU | fruit | 7.91 | 2.95E-23 | true | ** | Modulates mRNAs during fruit ripening and, hormone signaling |
| miR1916 | AUUUCACUUAGACACCUCAA | fruit | 3.82 | 0.04 | true | ** | - |
| miR1917 | AUUAAUAAAGAGUGCUAAAGU | fruit | 8.07 | 1.56E-25 | true | ** | - |
| miR1919a | ACGAGAGUCAUCUGUGACAGG | fruit | 3.22 | 1.45E-17 | true | ** | Functions in tomato fruit development |
| miR394 | UUGGCAUUCUGUCCACCUCC | fruit | 10.02 | 1.89E-76 | true | ** | - |
| miR395a | CUGAAGUGUUUGGGGGAACUCC | fruit | 4.01 | 0 | true | ** | Works in sulfur metabolism and transport, and miRNAs processing under sulfur stress |
| miR397 | AUUGAGUGCAGCGUUGAUGA | fruit | 3.82 | 0.03 | true | ** | Reported as stress responsive in plant development |
| miR398a-3p | UAUGUUCUCAGGUCGCCCCUG | shoot | 3.1839 | 5.24E-23 | true | ** | Plays in response of various stresses, and nutrient stress |
| miR398b-3p | UUGUGUUCUCAGGUCACCCCU | fruit | 4.82 | 0 | true | ** | Plays in response of various stresses, and nutrient stress |
| miR399 | UGCCAAAGGAGAGUUGCCCUA | fruit | 7.91 | 2.95E-23 | true | ** | Plays in tomato fruit development and maturation |
| miR5300 | UCCCCAGUCCAGGCAUUCCAAC | fruit | 3.24 | 3.52E-30 | true | ** |  |
| miR5301 | UGUGGGUGGGGUGGAAAGAUU | fruit | 2.4 | 9.37E-171 | true |  | - |
| miR5303 | UUUUUGAAGAGUUCGAGCAAC | fruit | 6.82 | 3.01E-12 | true | ** | - |
| miR6022 | UGGAAGGGAGAAUAUCCAGGA | fruit | 5.01 | 0 | true | ** | - |
| miR6023 | UUCCAUGAAAGAGUUUUUGGAU | fruit | 2.48 | 2.75E-12 | true | ** | Signal transduction and/or disease resistance in tomato |
| miR6024 | UUUUAGCAAGAGUUGUUUUACC | fruit | 6.75 | 0 | true | ** | Function on disease resistant protein |
| miR6024-3p | UUUUAGCAAGAGUUGUUUUCCC | fruit | 6.15 | 3.59E-08 | true | ** | Signal transduction and/or disease resistance in tomato  Function on disease resistant protein |
| miR6026 | UUCUUGGCUAGAGUUGUAUUGC | fruit | 3.12 | 2.18E-22 | true | ** | - |
| miR8007a-5p | AUGUGGCACUUUUCGGAUUUUGAG | fruit | 3.82 | 0.03 | true | ** | Function on photomorphogenesis repressor |
| miR8021 | AUUCAAGGCUCAAACUCGAGACCU | fruit/shoot | 3.82 | 0.037 | true | ** | - |

NTPM, normalized transcripts per million; Signature-TRUE, differentially expressed miRNAs; **, significant at *P* < 0.01. SFG, solanaceae fruit graft; SFC, solanaceae fruit control.

**TABLE S8. Significantly down-regulated evolutionary known miRNAs in grafted tomato**

| **miRNA** | **Sequence** | **Target tissue** | **log2- fold change** | ***P*-value** | **Signature** | **Sig-level** | **Key roles** |
| --- | --- | --- | --- | --- | --- | --- | --- |
| miR156a | UGACAGAAGAGAGUGAGCAC | fruit/shoot | -3.42 | 1.12E-24 | true | ** | Over expression results in fruit formation and yield  Tomato fruit ripening |
| miR156f-5p | CUGACAGAAGAGAGUGAGCA | fruit | -4.3 | 5.71E-13 | true | ** | Over expression results in fruit formation and yield  Tomato fruit ripening |
| miR156g | UGACAGAAGAUAGAGAGCAC | fruit | -1.53 | 4.98E-81 | true | ** | Over expression results in fruit formation and yield  Tomato fruit ripening |
| miR166a-5p | GGAAUGUUGUCUGGCUCGAGG | fruit | -3.32 | 0 | true | ** | Stress responsive and involved in plant development |
| miR166b | UCGGACCAGGCUUCAUUCCUC | fruit | -1.70 | 0 | true | ** | Stress responsive and involved in plant development |
| miR167a | UGAAGCUGCCAGCAUGAUCUGG | fruit | -2.75 | 1.31E-87 | true | ** | Targets genes involved in auxin signaling |
| miR172a | AGAAUCUUGAUGAUGCUGCAU | shoot | -1.27 | 0 | true | ** | Modulates mRNAs during fruit ripening and, hormone signaling |
| miR319a | UUGGACUGAAGGGAGCUCCCU | fruit | -1.22 | 5.71E-137 | true | ** | Target (MYB genes) involved in carbon fixation, gene expression and signal transduction |
| miR319b | UUGGACUGAAGGGAGCUCCU | fruit | -1.44 | 6.33E-73 | true | ** | Target (MYB genes) involved in carbon fixation, gene expression and signal transduction |
| miR398a-3p | UAUGUUCUCAGGUCGCCCCUG | fruit | -1.20 | 4.04E-40 | true | ** | Plays in response of various stresses, and nutrient stress |
| miR5303f | AUUUUUGGAGAAUCUGACACGGGU | fruit | -5.8 | 2.77E-10 | true | ** | - |
| miR5303g | AUAUUUUUGAAGAGUCUGAGCAAC | fruit | -2.23 | 1.69E-76 | true | ** | - |
| miR5303h | AACAUUUUUGAAGAGUCUGAGCAA | fruit | -1.46 | 8.93E-56 | true | ** | - |
| miR7997a | AUGCUGCUCGGACUCUUCAAA | fruit | -4.07 | 1.45E-25 | true | ** | - |
| miR7997c | AUAUUGCUCGGACUCUUCAAAAAU | fruit | -4.75 | 0 | true | ** | - |
| miR8039 | UUUCCUAUCUGAACUAUCACC | fruit | -7.38 | 6.12E-25 | true | ** | - |

NTPM, normalized transcripts per million; Signature-TRUE, differentially expressed miRNAs; **, significant at P < 0.01. SFG, solanaceae fruit graft; SFC, solanaceae fruit control.
